# The Mars 2020 Engineering Cameras and Microphone on the Perseverance Rover: A Next-Generation Imaging System for Mars Exploration

**DOI:** 10.1007/s11214-020-00765-9

**Published:** 2020-11-24

**Authors:** J. N. Maki, D. Gruel, C. McKinney, M. A. Ravine, M. Morales, D. Lee, R. Willson, D. Copley-Woods, M. Valvo, T. Goodsall, J. McGuire, R. G. Sellar, J. A. Schaffner, M. A. Caplinger, J. M. Shamah, A. E. Johnson, H. Ansari, K. Singh, T. Litwin, R. Deen, A. Culver, N. Ruoff, D. Petrizzo, D. Kessler, C. Basset, T. Estlin, F. Alibay, A. Nelessen, S. Algermissen

**Affiliations:** 1 Jet Propulsion Laboratory, California Institute of Technology, Pasadena, CA USA; 2 Malin Space Science Systems, San Diego, CA USA

**Keywords:** Mars, Remote sensing, Planetary exploration, Rovers, Cameras, Space exploration

## Abstract

The Mars 2020 *Perseverance* rover is equipped with a next-generation engineering camera imaging system that represents an upgrade over previous Mars rover missions. These upgrades will improve the operational capabilities of the rover with an emphasis on drive planning, robotic arm operation, instrument operations, sample caching activities, and documentation of key events during entry, descent, and landing (EDL). There are a total of 16 cameras in the *Perseverance* engineering imaging system, including 9 cameras for surface operations and 7 cameras for EDL documentation. There are 3 types of cameras designed for surface operations: Navigation cameras (Navcams, quantity 2), Hazard Avoidance Cameras (Hazcams, quantity 6), and Cachecam (quantity 1). The Navcams will acquire color stereo images of the surface with a $96^{\circ}\times 73^{\circ}$ field of view at 0.33 mrad/pixel. The Hazcams will acquire color stereo images of the surface with a $136^{\circ}\times 102^{\circ}$ at 0.46 mrad/pixel. The Cachecam, a new camera type, will acquire images of Martian material inside the sample tubes during caching operations at a spatial scale of 12.5 microns/pixel. There are 5 types of EDL documentation cameras: The Parachute Uplook Cameras (PUCs, quantity 3), the Descent stage Downlook Camera (DDC, quantity 1), the Rover Uplook Camera (RUC, quantity 1), the Rover Descent Camera (RDC, quantity 1), and the Lander Vision System (LVS) Camera (LCAM, quantity 1). The PUCs are mounted on the parachute support structure and will acquire video of the parachute deployment event as part of a system to characterize parachute performance. The DDC is attached to the descent stage and pointed downward, it will characterize vehicle dynamics by capturing video of the rover as it descends from the skycrane. The rover-mounted RUC, attached to the rover and looking upward, will capture similar video of the skycrane from the vantage point of the rover and will also acquire video of the descent stage flyaway event. The RDC, attached to the rover and looking downward, will document plume dynamics by imaging the Martian surface before, during, and after rover touchdown. The LCAM, mounted to the bottom of the rover chassis and pointed downward, will acquire $90^{\circ}\times 90^{\circ}$ FOV images during the parachute descent phase of EDL as input to an onboard map localization by the Lander Vision System (LVS). The rover also carries a microphone, mounted externally on the rover chassis, to capture acoustic signatures during and after EDL. The *Perseverance* rover launched from Earth on July 30th, 2020, and touchdown on Mars is scheduled for February 18th, 2021.

## Introduction

The National Aeronautics and Space Administration (NASA) Mars 2020 *Perseverance* rover launched from Earth on July 30th, 2020 and is scheduled to land on Mars on February 18th, 2021. The rover is designed to drive across the surface and collect samples of surface material for possible return to Earth by a follow-on mission (Farley et al. 2020). To help achieve this task the Mars 2020 Rover is equipped with a next-generation engineering camera imaging system that represents a significant upgrade over the engineering camera systems flown on previous missions. The Mars 2020 upgrades are focused on three key areas. The first area is rover surface operations, including activities such as rover driving, robotic arm operations, and science instrument operations. The second area includes documentation of sample processing and handling operations inside the rover Adaptive Caching Assembly (ACA). The third area includes documentation of the performance and operation of the Entry, Descent, and Landing (EDL) system. Collectively the *Perseverance* next-generation imaging system will improve the capabilities of the Mars 2020 mission and help to enhance the mission science return relative to previous missions. This paper describes the Mars 2020 engineering camera hardware and associated flight and ground software.

### Scope

#### Engineering Cameras

This paper describes the 16 engineering cameras on the Mars 2020 rover. For the purposes of this paper the Mars 2020 engineering cameras are divided into three groups, based on the three separate development teams that designed, built, and delivered the hardware to the Mars 2020 ATLO (Assembly, Test, and Launch Operations) team. The first group of cameras is comprised of nine cameras dedicated to surface operations and includes the Navigation cameras (Navcams, quantity 2), the Front Hazard Avoidance cameras (Front Hazcams, quantity 4), the Rear Hazard Avoidance cameras (Rear Hazcams, quantity 2), and the sample Cache camera (Cachecam, quantity 1). The second group is comprised of six cameras dedicated to EDL documentation and includes a Parachute Uplook Camera (PUC, quantity 3), a Descent stage Downlook Camera (DDC, quantity 1), a Rover Uplook Camera (RUC, quantity 1), and a Rover Downlook Camera (RDC, quantity 1). The third group includes the LCAM (quantity 1), dedicated to providing critical image data to the Lander Vision System (LVS) during the parachute descent phase of EDL. Data from all 16 cameras will be available for use by the *Perseverance* science and engineering teams during the mission. Data from the engineering cameras will also be archived in NASA’s Planetary Data System.

#### Science Cameras

In addition to the 16 engineering cameras described in this paper, there are seven cameras on the rover dedicated to specific scientific investigations. The Mastcam-Z cameras (quantity 2) acquire color stereo images with matching variable focal lengths using a zoom lens (Bell et al. 2020). The SuperCam camera uses a next-generation Remote Microscopic Imager (RMI), described in Maurice et al. (2020), to acquire context images for spectrometer observations. The PIXL (Planetary Instrument for X-ray Lithochemistry) instrument uses a Micro Context Camera (MCC) to acquire context images and images of projected laser fiducial markings (Allwood et al. 2020). The SHERLOC (Scanning Habitable Environments with Raman & Luminescence for Organics & Chemicals) instrument contains two cameras: the Advanced Context Imager (ACI) for context imaging, and the WATSON (Wide Angle Topographic Sensor for Operations and eNgineering) camera acquires image for general documentation and context (Bhartia et al. 2020). The MEDA SkyCam acquires images of the Martian sky as part of a larger atmospheric science instrument package (Rodriguez-Manfredi et al. 2020).

The Navcam and Hazcam cameras provide targeting support and context imaging for these science cameras. Images from the science cameras will be co-registered to the Navcam and Hazcam images using calibration data acquired during Mars 2020 ATLO.

#### Summary of Perseverance Imaging System

There are a total of 23 cameras on the *Perseverance* mission (16 engineering cameras and 7 science cameras). Table 1 lists the cameras and locations. Of the 23 cameras, 19 are rover-mounted and 4 cameras are mounted to the entry vehicle. Of the 19 rover-mounted cameras, 16 of the cameras are designed for use during the nominal surface mission.

**Table 1:** List of cameras on the Perseverance rover and entry vehicle

Camera name	Quantity	Location	Reference	Type
Navcam	2	Rover (mast)	Maki et al. (2020) (this paper)	Engineering
Front Hazcam	4	Rover (body)	Maki et al. (2020) (this paper)	Engineering
Rear Hazcam	2	Rover (body)	Maki et al. (2020) (this paper)	Engineering
Cachecam	1	Rover (internal)	Maki et al. (2020) (this paper)	Engineering
PUC^a^	3	Parachute structure	Maki et al. (2020) (this paper)	Engineering
DDC^a^	1	Descent stage	Maki et al. (2020) (this paper)	Engineering
RUC^b^	1	Rover (top deck)	Maki et al. (2020) (this paper)	Engineering
RDC^b^	1	Rover (body)	Maki et al. (2020) (this paper)	Engineering
LCAM^c^	1	Rover (body)	Maki et al. (2020) (this paper)	Engineering
Mastcam-Z	2	Rover (mast)	Bell et al. (2020)	Science
SuperCam RMI	1	Rover (mast)	Maurice et al. (2020)	Science
PIXL MCC	1	Rover (arm)	Allwood et al. (2020)	Science
SHERLOC ACI	1	Rover (arm)	Bhartia et al. (2020)	Science
SHERLOC WATSON	1	Rover (arm)		Science
MEDA SkyCam	1	Rover (top deck)	Rodriguez-Manfredi et al. (2020)	Science
Totals	23 total: 19 rover-mounted + 4 mounted to the entry vehicle			

^a^Discarded during rover landing event

^b^Commandable after landing, but not designed to withstand surface environment

^c^Not commandable after landing

### Background

#### Navcams, Hazcams, and Cachecam

The first Mars rover engineering cameras flew on the Mars Pathfinder *Sojourner* microrover (Moore et al. 1997). The *Sojourner* rover flew three body-mounted cameras for traverse assessment and documentation of vehicle state, augmented by a pair of pan/tilt mast-mounted stereo color cameras on the Mars Pathfinder lander (Smith et al. 1997). The *Spirit* and *Opportunity* rovers standardized these camera types into an integrated imaging system, with pan/tilt cameras (Navcams) and body-mounted cameras (Hazcams), as described in Maki et al. 2003. The same Navcam and Hazcam designs were carried forward to the Mars Science Laboratory (MSL) *Curiosity* rover, which flew identical copies of the MER cameras (Maki et al. 2012).

The MER engineering cameras were designed and developed as part of a single camera production effort with four Pancam cameras (Bell et al. 2003) and two Microscopic Imager cameras (Herkenhoff et al. 2003). All 20 of the MER cameras shared the identical electronics and detector design, with different lenses determining the camera type. The Mars Phoenix mission flew two MER flight spare camera electronics and detector assemblies as part of the Surface Stereo Imager camera system (Lemmon et al. 2008). The Mars Science Laboratory (MSL) program ran a second production run of build-to-print copies of the MER Navcams and Hazcams. A total of 14 of these cameras were flown on MSL (Maki et al. 2012). The Mars InSight mission flew two MSL flight spare cameras, slightly modified with color filter arrays added to the detectors (Maki et al. 2018). A third MSL flight spare unit will fly on the Mars 2020 Perseverance rover as part of the MEDA Skycam (Rodriguez-Manfredi et al. 2020).

A total of 36 individual flight unit cameras from the MER and MSL missions have flown to Mars as of this writing. With an additional MSL flight spare camera flying on the *Perseverance* rover in 2020, the era for the MER/MSL cameras is coming to a close. The original camera design from MER is now over 20 years old, and electronics parts obsolescence has precluded any additional production runs without significant redesigns. Additionally, significant advancements in electronics and detector designs have occurred since MER, opening up the possibility of improvements over the original designs.

In early 2013 an internal Mars 2020 project study was commissioned to examine the possibility of modernizing the MER/MSL camera designs. After a prototyping phase, the project baselined a set of new Navcam and Hazcam cameras in October 2014, along with Cachecam, a new camera type. Detailed design began in 2015, and the next-generation engineering cameras were delivered to the Mars 2020 ATLO in the summer of 2019. During hardware development the next-generation engineering camera task was referred to as the Enhanced Engineering Camera (EECAM) task. Once integrated onto the rover the engineering cameras assume the traditional ECAM name, along with the individual “cam” names of Navcams, Front Hazcams, Rear Hazcams, and the newly designed Cachecam.

The Mars 2020 Navcams and Hazcams offer three primary improvements over MER and MSL. The first improvement is an upgrade to a detector with 3-channel, red/green/blue (RGB) color capability that will enable better contextual imaging capabilities than the previous engineering cameras, which only had a black/white capability. The second improvement is that the *Perseverance* cameras have wider fields of view than previous versions, which improves the quality of mosaics and increases downlink efficiency. The third improvement is that the *Perseverance* cameras have finer pixel scale (mrad/pixel) and are able to resolve more detail than the MER/MSL cameras. All of the Navcam and Hazcam cameras for MER, MSL, and Mars 2020 were built at the Jet Propulsion Laboratory/California Institute of Technology, in Pasadena, CA.

The Mars 2020 rover carries a new camera type, the Cachecam, a fixed-focus imager dedicated to sample inspection and documentation. Cachecam images will be used by the sample operations teams to document and verify the processing of the sample material during caching operations. The same images will also serve to document the tube contents prior to sealing. The closest previous-mission analog to the Cachecam is the fixed-focus Microscopic Imager (MI) flown on the MER mission (Herkenhoff et al. 2003). The MER MI was mounted on a robotic arm and was placed into position by the arm for imaging. The Cachecam, also a fixed-focus imager, is hard mounted inside the rover chassis. A robotic arm inside the ACA brings sample tubes to the Cachecam for imaging operations. The Cachecam was built at the Jet Propulsion Laboratory/California Institute of Technology as part of the Navcam and Hazcam production run.

#### EDLCAMs

In 2014 the Mars 2020 project began to investigate options for improving knowledge of Entry, Descent, and Landing (EDL) system performance using a video imaging system to document key EDL events. Of particular interest were the parachute deployment, skycrane deployment, rover touchdown, and lander rocket plume dynamics. The proposed solution involved qualifying commercial off the shelf (COTS) hardware with an emphasis on low cost and ease of system integration. In June of 2015 the Mars 2020 project added the EDL cameras (EDLCAMs) to the project baseline. The EDLCAMs were delivered to ATLO in the fall of 2019.

The EDLCAM system includes four new camera types, along with a rover chassis-mounted microphone for recording audio. Three Parachute Uplook Cameras (PUCs) will monitor parachute deployment. A Descent stage Downlook Camera (DDC) will record the rover as it descends from the skycrane, while the Rover Uplook Camera (RUC) simultaneously records the descent stage as seen from the rover. The Rover Downlook Camera (RDC) will image Mars as the rover touches down onto Mars. Video and audio from the EDLCAM system will be relayed to Earth in the subsequent sols after the rover is safely on Mars. The EDLCAM system was built with commercially available hardware, slightly modified for use on Mars 2020, and integrated/tested at the Jet Propulsion Laboratory/California Institute of Technology.

#### LCAM

The first camera sent to Mars for descent imaging was the Mars Descent Imager (MARDI), which flew on the Mars Polar Lander (MPL) mission in 1998 (Malin et al. 2001). The MPL spacecraft was lost during EDL and no image data were returned (Casani et al. 2000).

In 2004 the MER Descent Motion Estimation System (DIMES) system returned images during EDL as part of a system to detect and remove excess horizontal velocity during EDL (Johnson et al. 2007). The DIMES system acquired three images per rover at a rate of one image every 3.75 seconds, using a modified Navcam camera as a descent imager (Maki et al. 2003). The DIMES system successfully determined the horizontal velocity on both vehicles and triggered a successful velocity reduction on Spirit (no correction was required on Opportunity).

In 2008 the Mars Phoenix mission flew a second version of the MPL MARDI to Mars, along with a microphone. Phoenix MARDI data were not acquired during EDL due to last-minute concerns that the data transfer from the camera to the lander might interfere with the EDL system.

In 2012 the MSL *Curiosity* Mars Descent Imager (MARDI) successfully acquired images during EDL (Malin et al. 2017). Although the *Curiosity* MARDI had heritage from the earlier MPL and Phoenix MARDI designs, the Phoenix experience motivated a different data handling architecture for MSL. Rather than relying on the spacecraft for the readout and storage of MARDI images, the camera included its own non-volatile flash memory, making data acquisition and storage independent of the spacecraft during EDL. The MSL MARDI was developed along with the MSL Mastcam (Malin et al. 2017) and MAHLI cameras (Edgett et al. 2012) and incorporates a number of other improvements relative to the MPL and Phoenix versions of MARDI: a larger format ($1600\times 1200~\text{pixels}$), color (Bayer pattern filter) detector, transform-based lossy image compression (Joint Photographic Experts Group, JPEG) and a higher frame rate ($\sim4~\text{frames/second}$). The *Curiosity* MARDI improved the frame rate by $15\times$ over the MER descent imager.

In 2016 the Mars 2020 project incorporated the Lander Vision System (LVS) system into the Mars 2020 EDL design. A key component of the LVS is the LVS Camera (LCAM), which acquires images of the surface during parachute descent. The LVS determines the vehicle location by acquiring and correlating LCAM images to an onboard reference map. The onboard map is generated using data from the Mars Reconnaissance Orbiter (MRO) Context camera (CTX, Malin et al. 2007) and preloaded onto the vehicle prior to EDL. The spacecraft uses the LVS localization to determine a landing target that avoids hazards identified a-priori using MRO HiRISE (McEwen et al. 2007) imagery. The spacecraft flies down to this safe target during the powered descent phase of EDL. LVS has heritage from the MER Descent Motion Estimation System (DIMES) system. The technique employed by the Mars 2020 LVS is called Terrain Relative Navigation (TRN).

The LCAM is required to acquire and send global shutter images immediately upon command by the LVS with low time latency. Because these timing requirements could not be met with any existing space-qualified flight imaging systems, Malin Space Science Systems (MSSS) was selected to develop, build, and test a new system. LCAM has heritage from the earlier MARDI designs, and also incorporates features of other MSSS imaging systems (Ravine et al. 2016).

## Instrument Objectives and Requirements

### ECAMs

#### ECAM Objectives

The high-level objectives and requirements of the *Perseverance* engineering cameras are largely unchanged from the original *Spirit* and *Opportunity* requirements. The Navcams are designed to survey the terrain around the rover with a $360^{\circ}$ field of regard by acquiring images from atop a pan/tilt mast mounted on the top deck of the rover. Navcam images are used for traverse planning, science target identification and selection, robotic arm operation, and rover auto-navigation. Additionally, the Navcams will document the state of the vehicle by acquiring images of rover hardware and determine the rover attitude in the local Mars frame by acquiring images of the sun. The Hazcams are designed to image the areas immediately fore/aft of the vehicle, particularly in areas that do not have Navcam coverage due to occlusion by the rover body. The Hazcams support robotic arm operation and rover navigation. They also support inspection of the front/rear wheels the contact interface between the wheels and the terrain. The Cachecam will document the material in the sample tubes during sample processing.

#### ECAM Requirements, Improvements over Previous Generations

The *Perseverance* engineering camera design upgrades were based on lessons learned from rover surface operations on Mars, including the *Spirit* rover mission, active from 2004–2010, the *Opportunity* rover mission, active from 2004–2018, and the *Curiosity* rover, active from 2012 to present. Three primary limitations of the original MER/MSL designs have emerged over the course of over 16 years of continuous rover operations on Mars. The first is that the MER/MSL Navcam field of view (FOV) is too narrow to efficiently image the Martian landscape around the rover. The second limitation is that the MER/MSL engineering cameras provide a limited capability for the assessment of vehicle state and Martian terrain due to lack of color information. The third limitation is that the low angular pixel scale of the MER/MSL engineering cameras limit blind drive designations to approximately 40–50 meters and provide a limited ability to assess the state of vehicle hardware. The original MER camera designs were designed for a 90-Sol nominal surface mission. By improving on the original designs the *Perseverance* engineering cameras will help to improve mission performance over predecessor missions.

#### Field of View

**Fig. 1:**
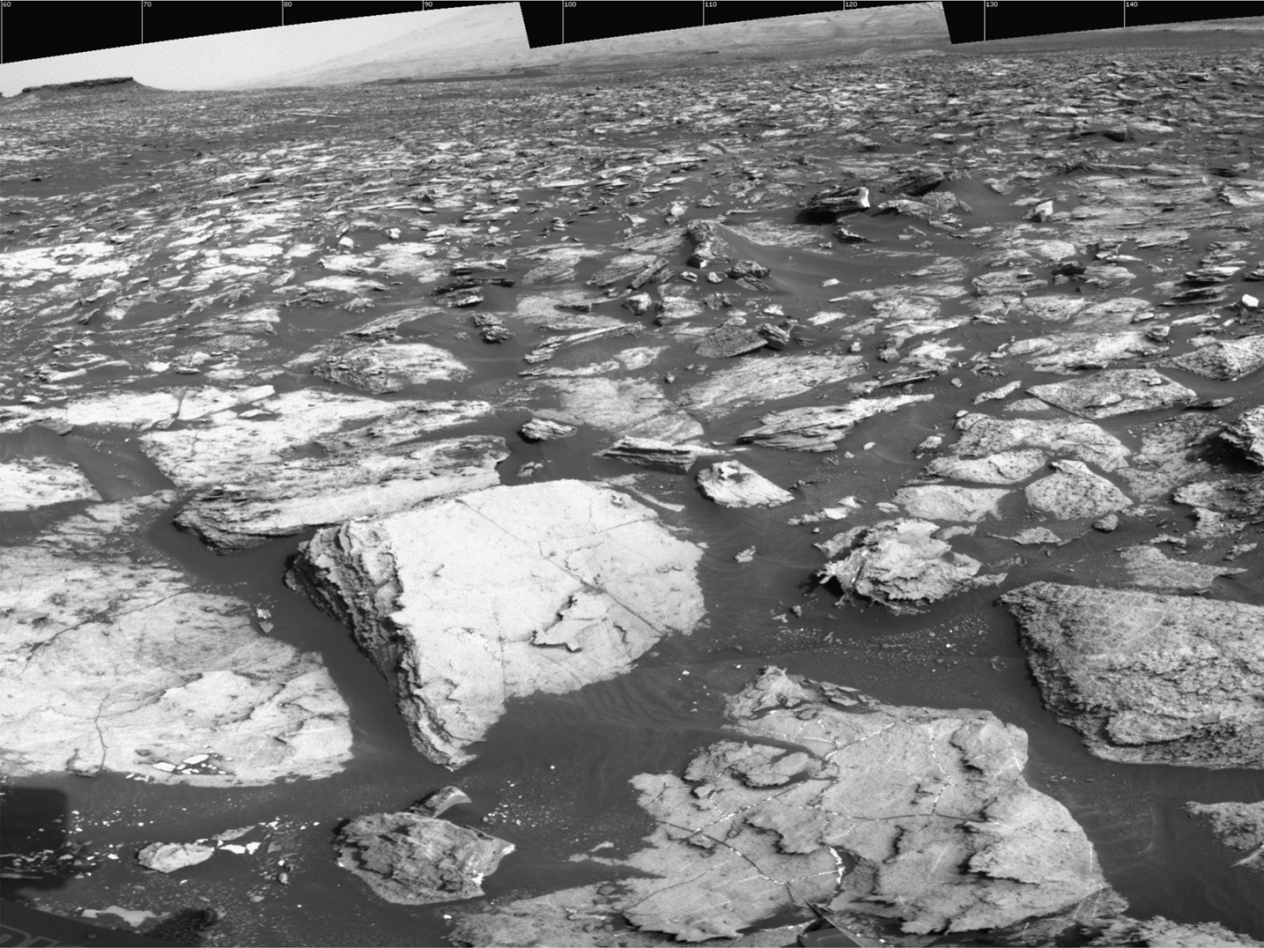
MSL Navcam mosaic. The mosaic as shown above covers a total field of approximately $90^{\circ}\times 70^{\circ}$, and required portions of 6 individual Navcam images ($\sim2.75$ images wide by $\sim1.75$ images high) to cover the field. The *Perseverance* Navcams will cover the same field of view in a single image. Reducing the image-to-image overlap in a mosaic improves the quality of a mosaic by reducing the number of seams. The reduction in overlap also reduces downlinked data volume by eliminating the redundant data in the overlap regions

The MER/MSL Navcam FOV is $45^{\circ} \times 45^{\circ}$ at 0.82 mrad/pixel. While a relatively wide field of view compared to Pancam ($16^{\circ}\times 16^{\circ}$ at 0.27 mrad/pixel, Bell et al. 2003) or Mastcam ($20^{\circ}\times 15^{\circ}$ at 0.218 mrad/pixel, Malin et al. 2017), the MER/MSL Navcam FOV is too narrow to allow simultaneous imaging of both the near field and far field terrain in a single image. A total of 10 MSL Navcam images must be acquired to cover the full $360^{\circ}$ field of regard. To cover both the near and far field terrain, two tiers of MSL Navcam panoramas (10 images wide × 2 images high) must be mosaicked together, creating image-to-image seams at the interfaces between the images (Fig. 1). Parallax effects between adjacent overlapping images within a panorama create imperfections within the final assembled mosaic.

The *Perseverance* Navcam field of view was chosen to be $90^{\circ}\times 70^{\circ}$. The $90^{\circ}$ horizontal FOV allows a $360^{\circ}$ Navcam panorama to be acquired with 5 overlapping images (compared to 10 overlapping Navcam images on MSL). Additionally, the $70^{\circ}$ vertical FOV enables a single Navcam image to cover the terrain from the area immediately near the rover all the way out to the horizon. The larger Navcam FOV also requires less data volume to cover the same terrain, saving between 5% and 10%, due to fewer overlap regions, as described in the next section (Field of View, Image-to-Image spacing, and Mosaic Coverage Efficiency).

The *Perseverance* Hazcam FOV requirement was expanded slightly to $>130^{\circ}$ horizontal, compared to $124^{\circ}$ in the MER/MSL Hazcam design, to enable better coverage of the rover wheels.

##### Field of View, Image-to-Image spacing, and Mosaic Coverage Efficiency

When imaging the terrain with a pan/tilt imaging system, the field of view of the camera is an important consideration in determining the angular (azimuth/elevation) spacing between adjacent images. This image-to-image spacing is also called the center-to-center spacing, i.e., the distance between the centers of adjacent images. In the ideal case where objects are infinitely far away from a perfect camera rotating about an idealized pinhole viewpoint, the center-to-center distance can theoretically be exactly equal to 100% of the FOV of the camera. In the case of a stereo pan/tilt system where the cameras rotate and translate about the pan/tilt axes, parallax effects require that the spacing between adjacent images be less than 100% of the FOV. Additionally, stereo coverage in a single stereo pair is not 100% due to parallax effects caused by the separation of the left/right eyes of a stereo pair. As a general rule, a center-to-center spacing of 80% of the camera FOV robustly ensures sufficient stereo coverage throughout an entire mosaic for virtually all scenes.

The spacing between the images in a mosaic can be expressed numerically with a spacing factor $\alpha $ variable multiplied by the FOV, where the value of $\alpha $ in this analysis ranges from 0.5 (50% spacing between images) to 1.0 (100% of the FOV) spacing between images). The mosaic coverage efficiency (*MCE*) can be expressed as the ratio of the total number of pixels acquired in a mosaic with spacing factor $\alpha $, including redundant pixels in the overlap regions, divided by the number of pixels in the same mosaic with no overlap ($\alpha =1$). For the case of a single mosaic image $(N=1)$, the MCE is 100%.

The equation for the *MCE* of a partial $360^{\circ}$ single tier mosaic containing $n$ images ($n \times 1$) each with areal coverage $A$ is:

$$ \textit{MCE}_{partial} = \frac{A_{1} +\alpha ( A_{2} + A_{3} + \cdots + A_{n} )}{A\times n} $$

where $A_{1}$, $A_{2}$, $A_{3}$, etc. are the individual areal coverages of each image in the mosaic (ignoring parallax effects) and $\alpha $ is the spacing factor.

Because the areal coverages are the same, the above equation simplifies to:

$$ \textit{MCE}_{partial} = \frac{1+\alpha (N-1)}{N} $$

In the case of an idealized full $360^{\circ}$ mosaic, ideally spaced so that $n$ images fit symmetrically into a $360^{\circ}$ panorama and the last ($n$th) image overlaps the first, the *MCE* is:

$$ \textit{MCE}_{full\ 360} = \frac{A_{1} +\alpha ( A_{2} + A_{3} + \cdots + A_{n-1} )+ A_{n} (2\alpha -1))}{A\times n} $$

**Fig. 2:**
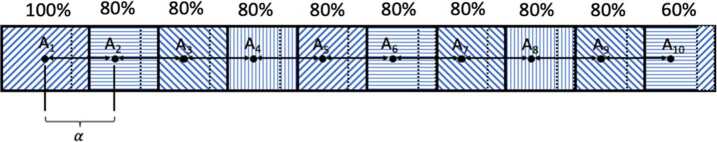
Individual image coverage efficiency of images within a $10 \times 1$, 360-degree mosaic with spacing factor $\alpha =0.8$. The total cumulative mosaic coverage efficiency is shown in Fig. 3

In the above equation, the first image, $A_{0}$ has 100% efficiency (no overlap), while subsequent images have an efficiency of $\alpha $. The very last image in a 360-degree mosaic is the least efficient, with an efficiency of $2\alpha -1$ due to the fact that it is wrapping around to the first image in mosaic and thus covering redundant areas on both sides of the image, as shown in Fig. 2. This is equivalent to all of the images having an efficiency equal to $\alpha $, and the above equation can be simplified to show that the MCE of a full 360-degree mosaic is equal to the spacing factor:

$$ \textit{MCE}_{full\ 360} =\alpha $$

One interesting result from this analysis is that the *MCE* for an idealized, complete $360^{\circ}$ single-tier mosaic is equal to the spacing factor $\alpha $ regardless of the FOV of the camera. A small FOV camera will have many seams, but as long as the panorama covers $360^{\circ}$ it will have the same mosaic coverage efficiency as a large FOV camera. Conversely, for partial panoramas, a camera with a wider FOV will generally give higher mosaic coverage efficiencies than smaller FOV cameras. The above analysis is idealized, but the results are generally the same, even for non-idealized panoramas.

**Fig. 3:**
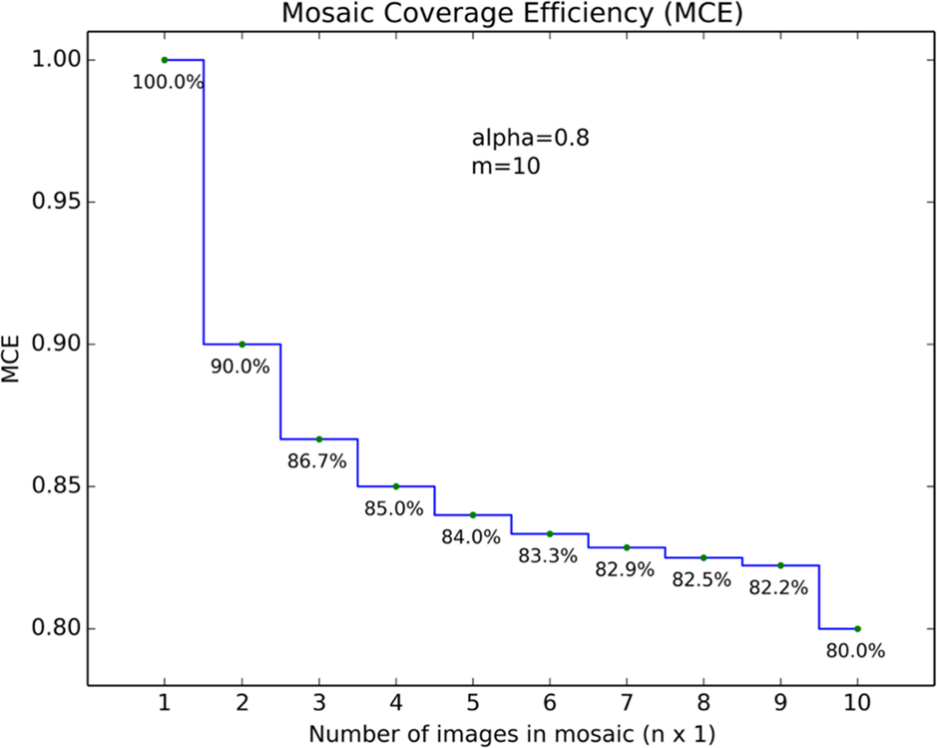
Mosaic coverage efficiency for an MSL $10\times1$ Navcam $360^{\circ}$ mosaic, plotted as a function of the number of images (horizontal , $\alpha =0.8$, $m=10$). The variable $m$ represents the number of images required for a full $360^{\circ}$ mosaic

**Fig. 4:**
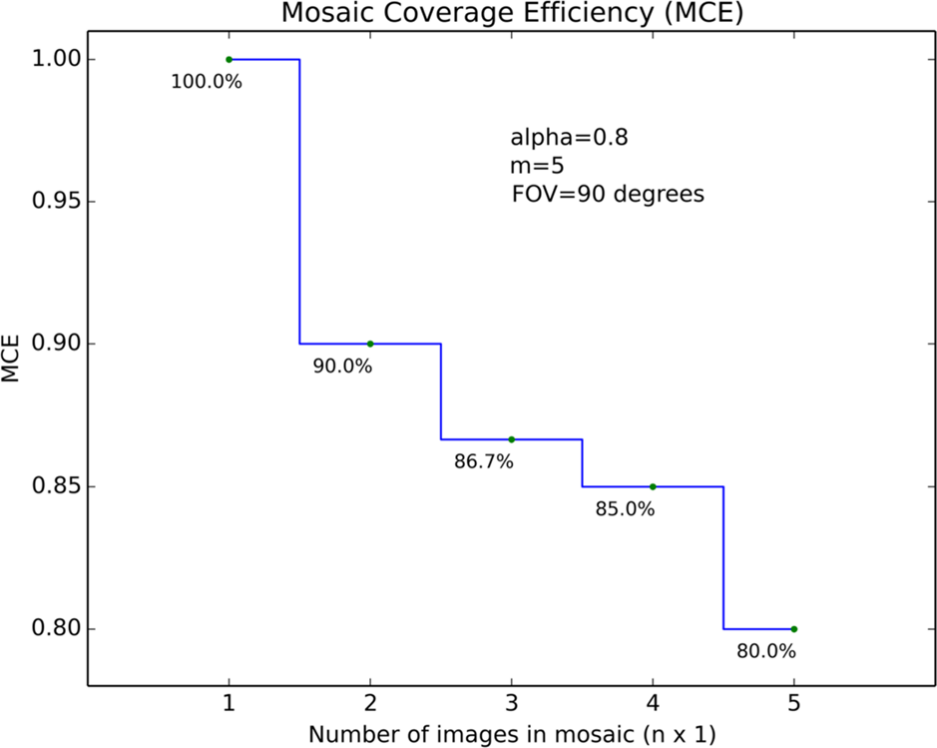
Mosaic coverage efficiency for a *Perseverance* $5\times1$ Navcam $360^{\circ}$ Navcam mosaic, plotted as a function of the number of images (horizontal , $\alpha =0.8$, $m=5$). The variable $m$ represents the number of images required for a full $360^{\circ}$ mosaic

**Fig. 5:**
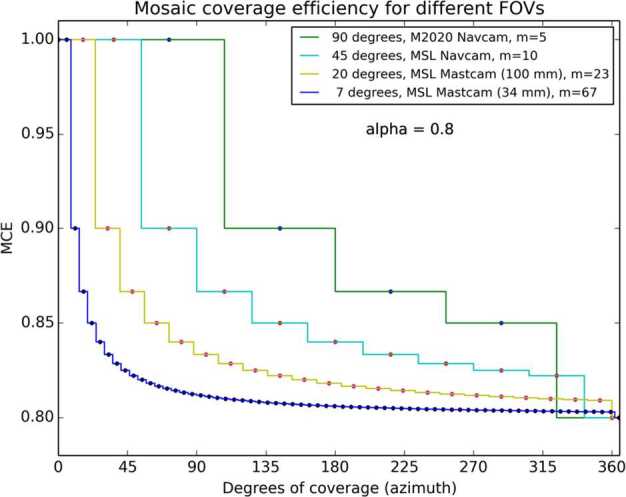
The MCE for several camera types, plotted as a function of azimuth. For coverage less than $360^{\circ}$, a wider FOV camera will have a higher mosaic coverage efficiency than a camera with a smaller FOV. The variable $m$ represents the number of images in a full $360^{\circ}$ mosaic

The plot in Fig. 3 shows how the mosaic coverage efficiency for a single tier MSL Navcam mosaic starts at 100% for the first image, decreases to 90% for the $2\times1$ mosaic, 86.7% for the $3\times1$, and so on, finally dropping to $\alpha $ as the mosaic forms a complete $10\times1$, $360^{\circ}$ panorama. Figure 4 shows a *MCE* plot for a $5\times1$, $360^{\circ}$ Mars 2020 Navcam panorama. With the same $\alpha $ value of 0.8, the MCE values for the Mars 2020 Navcam are identical to the MSL Navcam values for the first 4 images. The fifth image in the Mars 2020 Navcam completes the $360^{\circ}$ panorama, which drops the MCE for the full $5\times1$ panorama to 0.8, while the efficiency of the $5\times1$ MSL Navcam panorama is 84%. The difference however is that the MSL Navcam panorama only covers $180^{\circ}$, while the Mars 2020 panorama covers the full $360^{\circ}$. This is more easily shown in Fig. 5, which compares the MCE values of four different camera FOVs.

By increasing the FOV of the Navcam from $45^{\circ}$ (MSL) to $90^{\circ}$ (*Perseverance*), the mosaic coverage efficiency improves across the whole range of azimuths. Of particular interest are the partial panoramas covering between $90^{\circ}$ and $180^{\circ}$, which are often acquired as part of a drive direction or arm workspace images. For these types of panoramas, the mosaic coverage efficiency for the *Perseverance* Navcams is typically 90%, compared to $\sim85\%$ for the corresponding MSL Navcam. For panoramas less than $90^{\circ}$, the *Perseverance* MCE values are 100%, compared to the comparable Navcam MCE values of $\sim90\%$.

The higher *Perseverance* Navcam efficiency results in a typical data volume savings of approximately 5% when compared to the MSL Navcams. When tallied over the entire nominal mission, such a data volume savings corresponds to the equivalent of several thousand Navcam images.

The MCE analysis can be extended into two dimensions with a similar conclusion: cameras with wider fields of view use downlink bandwidth more efficiently than narrower FOV cameras when acquiring partial panoramas.

In addition to better mosaics with higher coverage efficiency, the wider *Perseverance* Navcam FOV means that the onboard navigation software (visodom and autonav) only needs a single stereo Navcam image acquisition rather than the 3 stereo Navcam images required on MSL. This allows the *Perseverance* Navcams to be pointed once and left there during a drive, saving actuator usage, Remote Sensing Mast (RSM) slew time, and image acquisition time. This time savings can instead be spent directly on rover driving, which improves drive distance per sol.

#### ECAM Pixel Scale

**Fig. 6:**
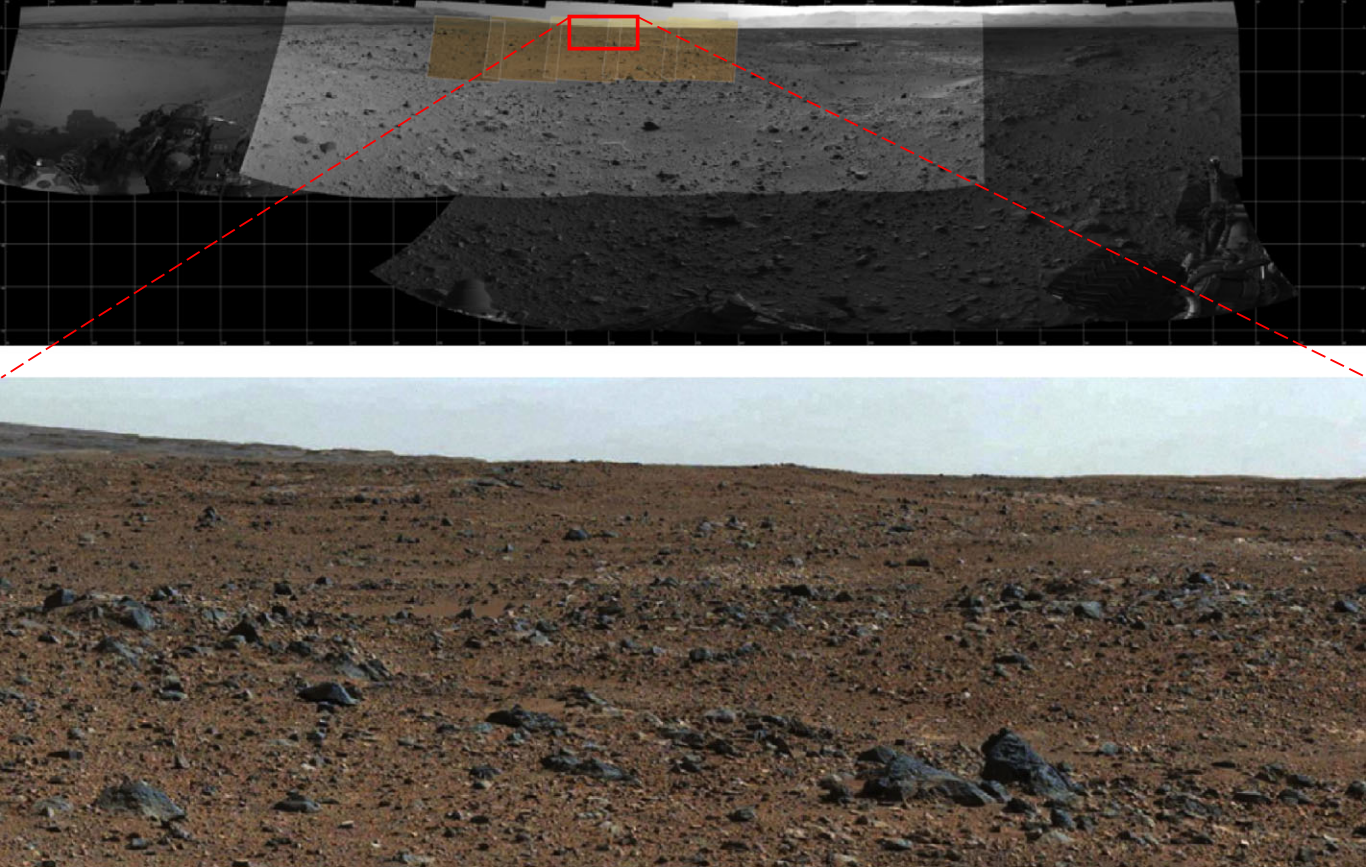
Drive direction imaging with the MSL Navcams (greyscale, 0.8 milliradians/pixel) and the left Mastcam (color, $\sim0.2~\text{mrad/pixel}$, inset in top image, and bottom image). The *Perseverance* Navcams have a pixel scale of $\sim0.3~\text{mrad/pixel}$

The MER/MSL Navcams have a pixel scale of 0.8 milliradians/pixel at the center of the FOV, and the MER/MSL Hazcams have a pixel scale of 2.1 milliradians/pixel at the center of the FOV. While these pixel scales are sufficient for basic context and planning, the limited MSL Navcam pixel scale prevents drive planning beyond approximately 50 meters on MSL, depending on the terrain. Additionally, the ability to resolve details of rover hardware with the MSL Navcams is often insufficient, particularly when imaging the turret hardware on the robotic arm. During surface operations the *Curiosity* rover engineering team often requests images from the Mastcams, with a pixel scale of 0.218 mrad/pixel, for drive planning (Fig. 6) and engineering inspection of hardware. The *Perseverance* Navcams and Hazcams were required to have pixel scales of $\leq0.4~\text{mrad/pixel}$ to allow better drive planning and hardware inspection capabilities.

##### ECAM Color

**Fig. 7:**
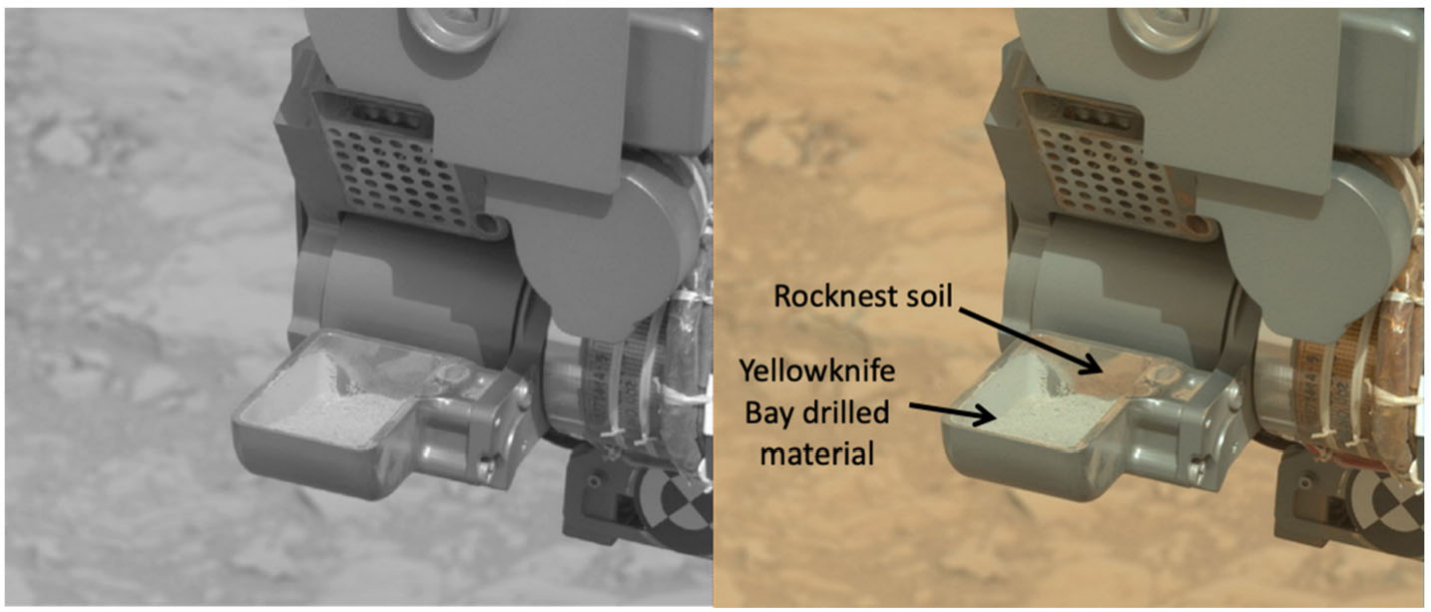
Distinguishing two types of Martian material against metallic surfaces is challenging when using only luminance information (left), while the same assessment is relatively straightforward with 3-channel color (right). This image is from the MSL Mastcam (Malin et al. 2017). All of the *Perseverance* engineering cameras are color cameras

The MER/MSL engineering cameras acquire single-channel greyscale images, with a visible bandpass covering 600–800 nm. The lack of color information often hampers the ability of ground operators to assess the state of the vehicle during surface operations. One particularly challenging area involves assessing the level of dustiness of rover hardware. When assessing the quantity of dust, soil, and drilled rock material against anodized aluminum and other shiny metallic surfaces, the use of grayscale images for this assessment is challenging even for experienced human operators (Fig. 7).

Color also improves the ability of operators and algorithms to distinguish between terrain types, in particular areas that contain dust and soil versus areas that contain more rocky materials. Because Martian soil/dust is a distinct yellowish brown in color (Huck et al. 1977; Maki et al. 1999), it is readily identified in color images in ways that are not possible with greyscale images (Maki et al. 2018). Because of this advantage over grayscale, all of the *Perseverance* engineering camera designs were required to have color (RGB) detectors.

#### Other ECAM Design Considerations

As part of the *Perseverance* engineering camera design effort, all previous engineering camera requirements from MER/MSL were either to be met or exceeded. During the design phase, the MER/MSL cameras were used as the minimum performance threshold, with the performance of the new ECAMs being capabilities-driven above that threshold, within a project-defined cost constraint.

##### Interface

As part of the upgrade to the next-generation designs, one of the key requirements on the *Perseverance* engineering camera design was that it maintain near 100% compatibility with the heritage MER/MSL interface, including electronics interface, data, power, and mechanical interface. This was driven by the fact that the engineering camera interface card inside the rover body is a build-to-print copy of the MSL card. Memory inside the *Perseverance* Rover Compute Element (RCE) is also unchanged. This constraint drove the design of the *Perseverance* ECAM tiling scheme, discussed in detail in Sect. 3. The *Perseverance* ECAM design team was given the goal of designing a camera that was as close to “plug and play” compatible with the MSL and MER interfaces as possible.

##### Thermal

The MER/MSL camera thermal design isolated the camera head electronics from the focal plane array electronics by placing them in separate housings/boxes, in an effort to minimize the amount of energy required to heat the cameras. During surface operations use however, the energy required to heat the engineering cameras on MER/MSL was negligible. In fact, the MER/MSL engineering camera thermal design was so efficient that it created overheat risk due to the fast warmup of the cameras when the camera heater switches were turned on. Because of this lesson learned the *Perseverance* ECAM thermal design combines the camera head electronics and focal plane array electronics into a single mechanical package, thermally isolated from the bracket/mounting interface using titanium standoffs. Unlike the focal planes used in the MER/MSL cameras, the *Perseverance* ECAM focal planes are operated in the same temperature range as the main camera electronics. The electronics boards for the *Perseverance* ECAMs are thermally coupled to the camera housings. This thermally isolates the electronics from the mounting brackets and reduces the amount of energy required to heat the electronics to operating temperature. A relatively small amount of energy is required to energize the film heaters on the exterior of the camera housings, which heats the enclosure and ultimately the electronics.

### EDLCAMs

#### EDLCAM Objectives

The objectives of the EDLCAM imaging system are to record key events during EDL. The first event, parachute deployment, will be recorded by three Parachute Uplook Cameras (PUCs). The PUCs will record the deployment, inflation, and operational dynamics of the parachute from just prior to mortar fire until backshell separation. The second event, sky crane deployment, will be recorded by the Descent stage Downlook Camera (DDC) and the Rover Uplook Camera (RUC). The DDC will record rover dynamics and ground interaction with the sky crane plume through touchdown. The RUC will record the descent stage dynamics through touchdown as seen from the rover and will also capture the flyaway of the sky crane. The sixth camera, the Rover Downlook Camera (RDC), will capture the rover dynamics and plume interaction with the ground as the rover touches down onto Mars.

**Fig. 8:**
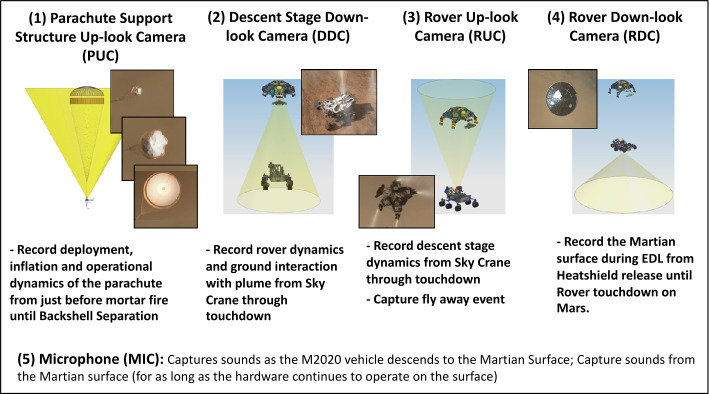
Location, approximate boresight location and direction, and notional image examples for the EDLCAMs

**Fig. 9:**
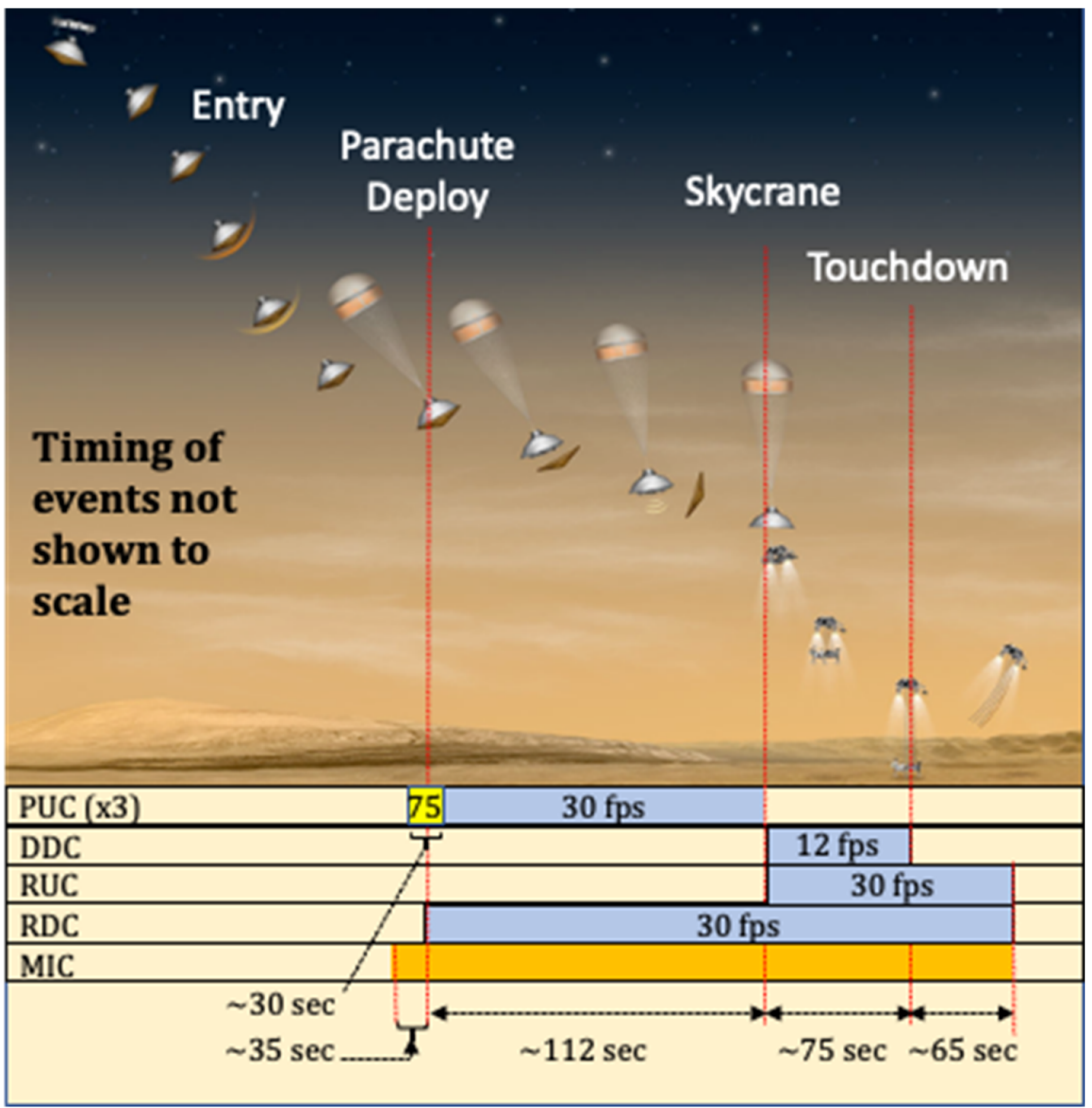
EDLCAM recording sequence, showing the approximate coverage of the data acquisition phases of the EDLCAM system. The acquisition frame rates of the cameras are listed in frames per second (fps), and the acquisition durations are listed on the bottom. The PUC frame rate listed in the yellow box is 75 fps

Additionally, the EDLCAM system includes a microphone, designed to capture acoustic events from parachute deploy to touchdown. Figure 8 shows the locations and notional fields of view of the cameras. The EDLCAM recording phases are shown in Fig. 9.

#### EDLCAM Requirements

The EDLCAMs were developed with a minimal number of key requirements. The first requirement was that the EDLCAMs must not interfere with the flight system during the critical EDL phase of the mission. This requires that the EDLCAM imaging system have zero or near zero interaction with the MSL-heritage flight system elements during EDL. Data for the EDLCAMs must be stored offline from the main rover computer during EDL, to be retrieved after the vehicle is safely on the surface, much in the same way that the MSL MARDI cameras operated (Malin et al. 2017). The EDLCAM system was specifically designed to not impose significant changes to heritage hardware or software on the MSL flight system. The frame rates for the camera video were driven by a minimum frame rate requirement of 30 frames per second (fps) for parachute imaging. All other EDLCAM performance attributes are capabilities driven. The EDL camera system was categorized as a technology demonstration during development, and the system uses a significant amount of commercially available hardware, qualified and modified for EDL use.

### LCAM

#### Objectives

**Fig. 10:**
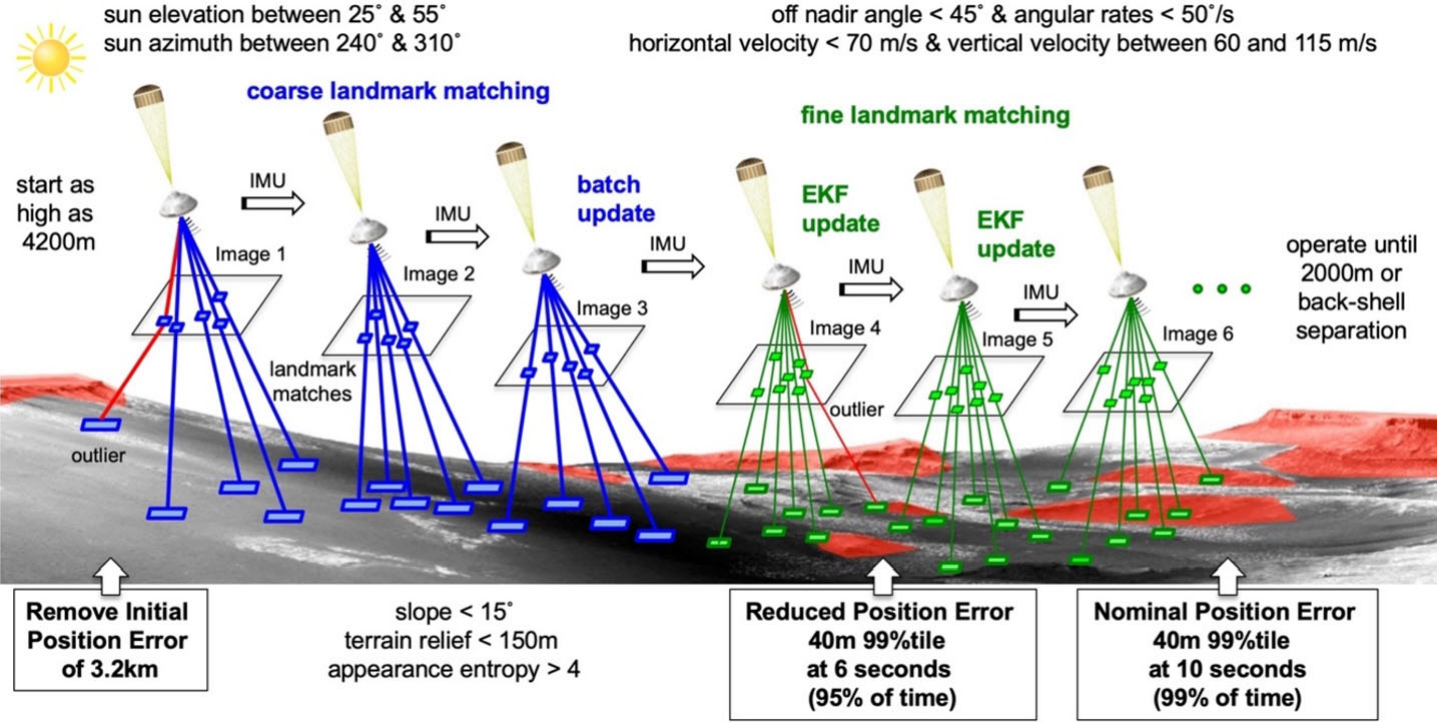
LCAM images are used by LVS to match features against a basemap. These feature matches are sent to an Extended Kalman Filter (EKF) that estimates the vehicle position. The vehicle attitude is propagated by an Inertial Measurement Unit (IMU). For details on the LVS see Johnson et al. 2017. Figure from Johnson et al. 2017

LCAM will acquire images during the parachute descent stage of EDL for the LVS. Figure 10 shows how LCAM images are matched to a basemap during EDL.

#### LCAM Requirements

The key LCAM requirements were for a field of view $90^{\circ}$ by $90^{\circ}$, a format of 1024 by 1024 pixels, a detector with a global shutter, a frame latency of less than 100 ms between the camera image trigger and the last pixel output of the image, a frame rate of up to 2 Hz, and SNR of $>80{:}1$ with $\sim1$ millisecond exposure time under Martian illumination conditions.

## Instrument Description and Performance

### ECAMs

All of the *Perseverance* engineering cameras share the same detector and camera body designs. This approach helps reduce development costs and greatly simplifies integration and test activities, the software and image processing architecture, and camera operation. Table 2 lists the characteristics of the Navcam, Hazcam, and Cachcam cameras. A total of 23 cameras were built in the *Perseverance* engineering camera production run, including 9 flight model units (FM), 9 engineering model (EM) units, 4 flight spare (kits), and one qualification model (QM). The ECAM lenses were designed and manufactured by Jenoptik.

**Table 2:** Perseverance Navcam, Hazcam, and Cachecam characteristics

	Navcam	Hazcam	Cachecam
Horizontal FOV	96^∘^	136^∘^	$\sim50~\text{mm}$ diameter circle at the plane of focus
Vertical FOV	73^∘^	102^∘^	
Diagonal FOV	120^∘^	172^∘^	
Focal ratio	f/12	f/12	N/A
Focal length	19.1 mm	14.0 mm	0.51 magnification (measured)
Best focus	3.5 meters	0.9 meters	$\sim140~\text{mm}$ below illuminator mirror and $\sim130~\text{mm}$ below exit aperture window
			Depth of field $\pm5~\text{mm}$
Pixel scale (center of FOV)	0.33 mrad/pixel	0.46 mrad/pixel	$\sim12.5~\text{microns/pixel}$ at plane of focus
Mass (per camera)	411 grams	498 grams	397 grams (camera)
			398 grams (vision station)
Volume	$74~\text{mm}\times 88~\text{mm}\times 125~\text{mm}$	$74~\text{mm}\times 88~\text{mm}\times 140~\text{mm}$	$74~\text{mm}\times 88~\text{mm}\times 143~\text{mm}$
Stereo baseline	42.4 cm	Front: 24.8 cm	N/A
		Rear: 93.4 cm	
Angle between left/right boresights	<0.4^∘^ (parallel)	Front: 20^∘^	N/A
		Rear: <1^∘^ (parallel)	
Boresight mounting orientation	Mounted to pan/tilt RSM, left/right camera boresights are parallel	Front: 28^∘^ below nominal horizon, left/right cameras are canted outward by 10^∘^ each	Pointed downward inside of the ACA. Samples are brought up to the vision station by the SHA (Sample Handling Assembly)
		Rear: 45^∘^ below the nominal horizon, left/right camera boresights are parallel	
Height above nominal surface	∼1.98 meters when viewing horizon	0.73 meters	0.73 meters

Detector characteristics	
Pixel format	5120 × 3840
Pixel pitch	$6.4~\upmu \text{m}\times 6.4~\upmu \text{m}$
Optical format	Full frame ($32.77~\text{mm}\times 24.58~\text{mm}$)
Pixel type	Global shutter with correlated double sampling
Shutter type	Pipelined global shutter
Full well	15,000 e-
Pixel dark noise	8 e- RMS
Conversion gain	0.24 DN/e-
Readout time	237 milliseconds
Exposure time (commandable)	410.96 microseconds to 3.277161 seconds, in 50 microsecond steps using autoexposure algorithm from Maki et al. (2003)
Responsivity	0.29 A/W @ 55 nm with microlenses
Maximum SNR	41.8 dB
Dynamic Range	66 dB
Color Filters	RGB Bayer color filter array (CFA)
QE	64.5% @ 55 nm with microlenses
Camera interface to rover	
Command and data interface	LVDS
Protocol	MER/MSL
Power Input	+4.3 Volts to +5.9 Volts
Data Rate	500,000 pixels/second
Power	3 Watts (single camera, imaging mode)
	1 Watt (single camera, idle)
Memory	1 Gbit SDRAM
FPGA	MicroSemi Rad-Tolerant ProASIC3
Radiation Tolerance	20 kRad TID, RDF = 2
Temperature Range	$-55~^{\circ}\text{C}$ to $+50~^{\circ}\text{C}$ (operational)
	$-135~^{\circ}\text{C}$ to $+70~^{\circ}\text{C}$ (survival)

#### Detector

**Fig. 11:**
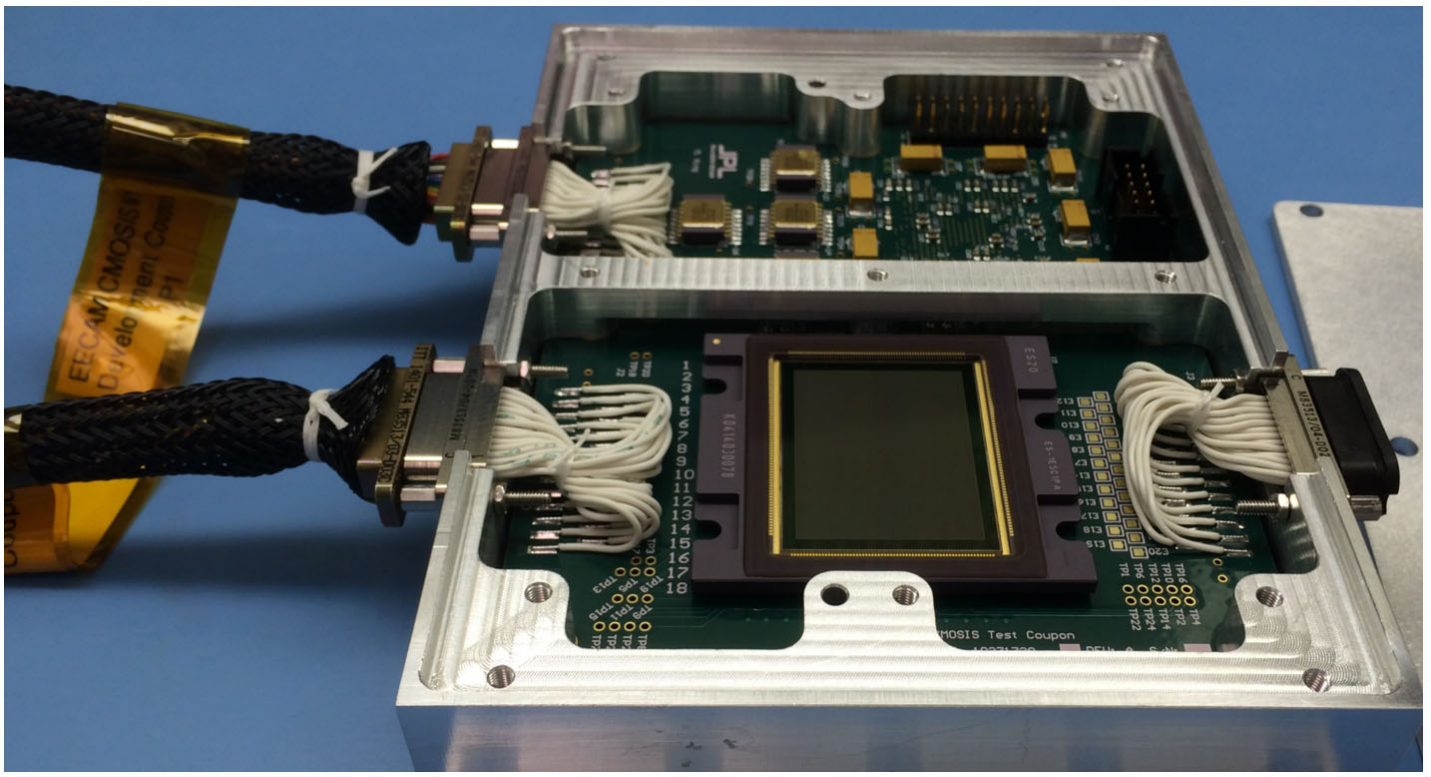
A CMV-20000 detector undergoing prototype testing in 2014 at JPL. The active imaging area is $32.77~\text{mm}\times24.58~\text{mm}$

The *Perseverance* engineering cameras use a CMV-20000 detector (Fig. 11), a global shutter CMOS (Complimentary Metal Oxide Semiconductor) device with $5120\times 3840$ pixels, built by AMS (formerly CMOSIS). The image array has $6.4~\text{micron}\times 6.4~\text{micron}$ sized pixels, digitized at 12 bits/pixel. The detector has a full-frame optical size of 32.77 mm by 24.58 mm with a Bayer pattern color filter array (Bayer 1976). The CMV-20000 was chosen due to the large optical format, the relatively high dynamic range, and high frame rate capability. The *Perseverance* engineering cameras have a commandable exposure time range of 410.96 microseconds to 3.277161 seconds, in 50 microsecond steps. The detector has been radiation tested to 20 kRad, RDF (Radiation Design Factor) $=2$ and meets total dose mission performance requirements.

#### Electronics

**Fig. 12:**
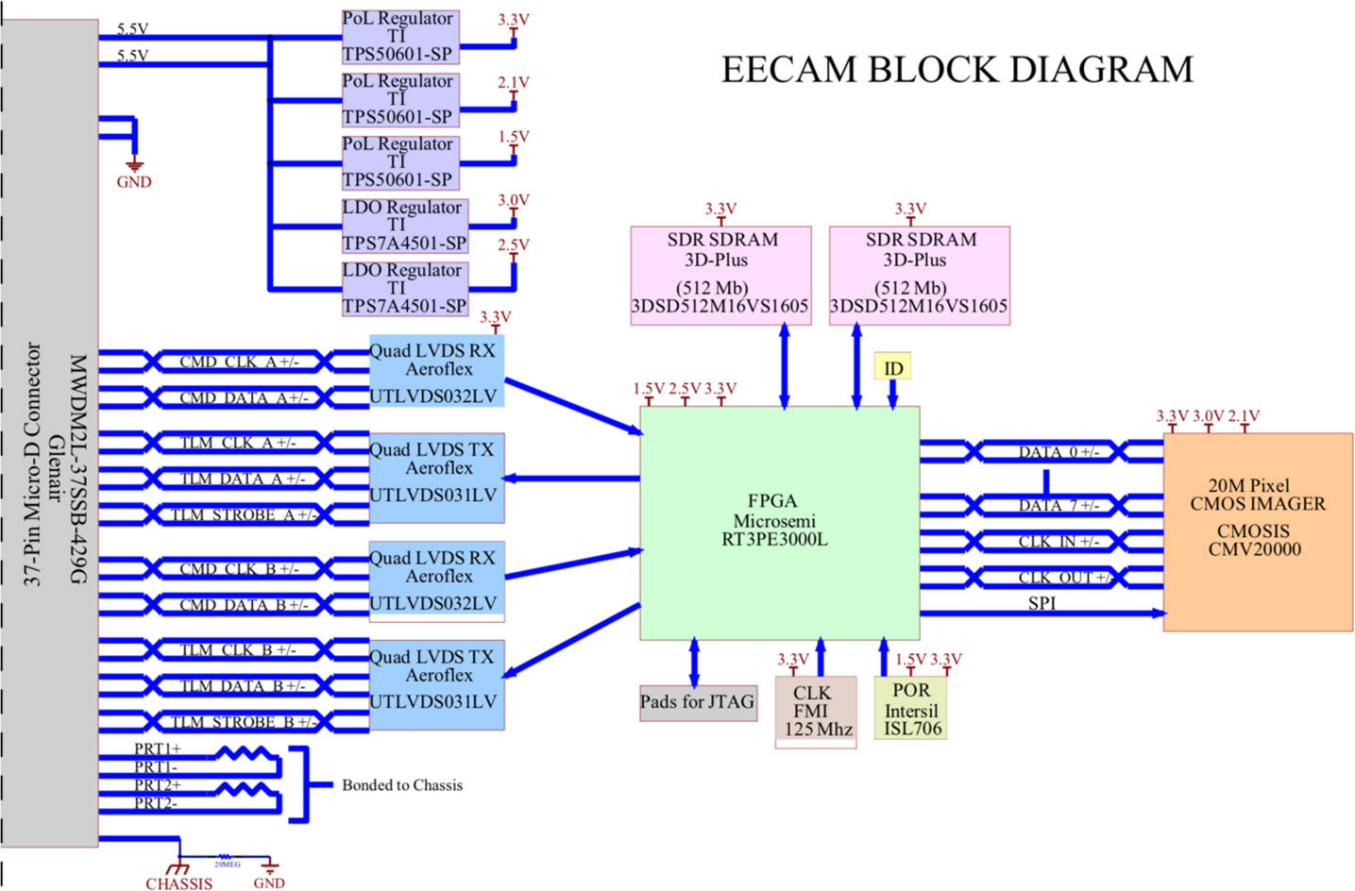
*Perseverance* camera electronics block diagram

The camera electronics consist of 3 electronics PWBs (printed wiring boards) connected via rigid flex polyimide film layers built into the inner layers of the boards. This integrated approach simplifies the design and manufacturing, and follows the approach used on MER and MSL. The three PWBs are folded into the camera body mechanical housing. The electronics are connected to the rover interface using a 37-pin microD connector. Figure 12 shows the functional block diagram for the ECAM electronics. The pixel transfer rate between the camera and the RCE was increased to 500,000 pixels/second without changing the heritage LVDS clock rate by inserting less space between pixel waveforms. The MER/MSL pixel transfer rate is $\sim200{,}000$ pixels per/second.

#### Hazcams

**Fig. 13:**
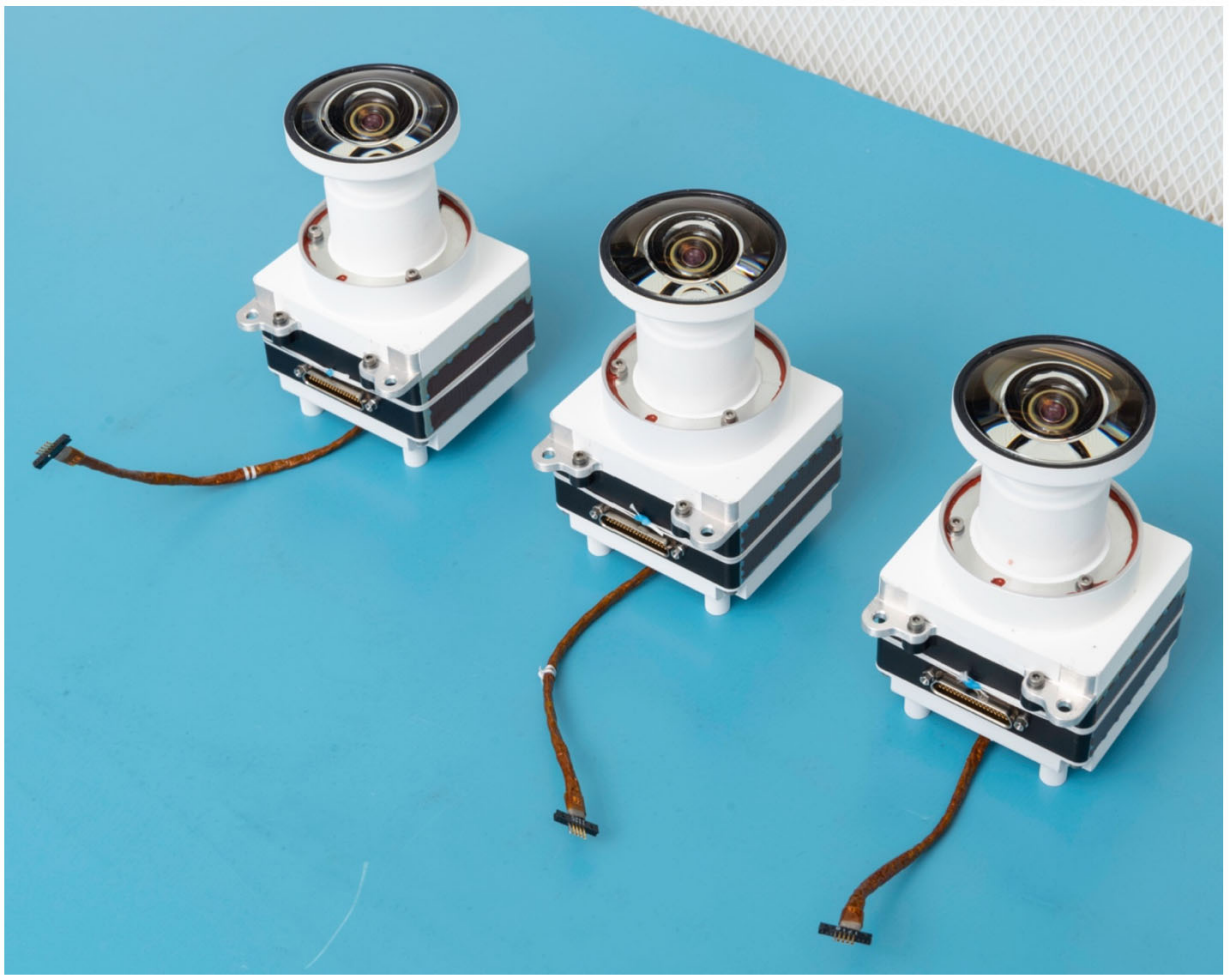
Flight Hazcam cameras

The Hazcam lenses have a focal ratio of f/12 and a best focus distance of approximately 0.9 meters, optimized for the robotic arm workspace and views of the rover wheels. Each Hazcam lens assembly contains 10 individual lens elements and a fused silica UV and IR blocking filter mounted inside the lens assembly between the third and fourth elements. The cameras are body-mounted to the fore and aft of the rover. The FOV of the Hazcams is $136^{\circ}$ (horizontal) by $102^{\circ}$ (vertical), with a $173^{\circ}$ diagonal. Figure 13 shows a set of flight Hazcams.

##### Front Hazcams

**Fig. 14:**
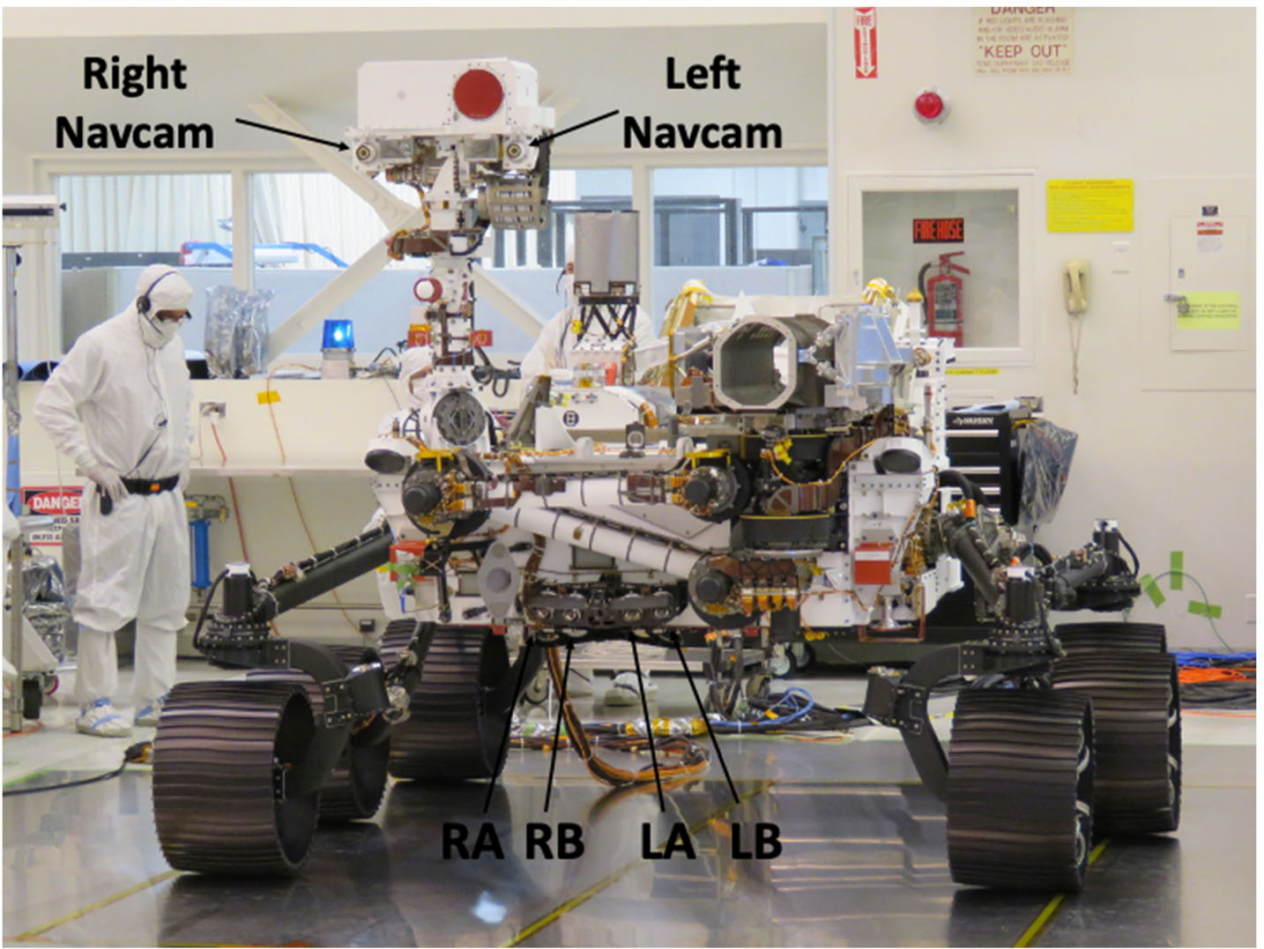
Perseverance Front Hazcams. $\text{L} = \text{left}$, $\text{R} = \text{right}$, $\text{A} = \text{RCE-A}$, $\text{B}=\text{RCE-B}$. Also shown are the Left/Right Navcams

There are four Hazcams hard-mounted to the rover front chassis (Fig. 14). The Front Hazcams form two stereo pairs: one pair is connected to RCE-A, and the second pair is connected to RCE-B. The Front Hazcam stereo baseline for each pair is 24.8 cm; the A/B cameras are interleaved to maximize the stereo baseline between the left and right cameras. The Front Hazcams are pointed downward $28^{\circ}$ from the nominal horizon to allow better coverage in the area immediately in front of the rover. Additionally, the left and right Front Hazcam boresights are each angled outwards by $10^{\circ}$ (the left cameras are angled $10^{\circ}$ to the left and the right cameras are angled $10^{\circ}$ to the right) to allow better viewing of the rover wheels.

The Front Hazcams have a sun visor mounted above the cameras to prevent sunlight from falling directly onto the lenses. This helps reduce lens flare and other scattered light artifacts. To maximize sun shading during late afternoon imaging the visor extends into the top of the camera FOV slightly. These upper regions of the FOV will typically be cropped (subframed) out of the image before being sent for downlink.

The Front Hazcams are protected during EDL by a camera cover assembly. The camera covers have transparent Lexan windows, which allow useable images to be acquired while the covers are closed. The covers are released shortly after touchdown on Mars using a non-explosive actuator (NEA). Once released, the covers flip open using a spring loaded-mechanism and stay in the open position for the remainder of the mission.

##### Rear Hazcams

**Fig. 15:**
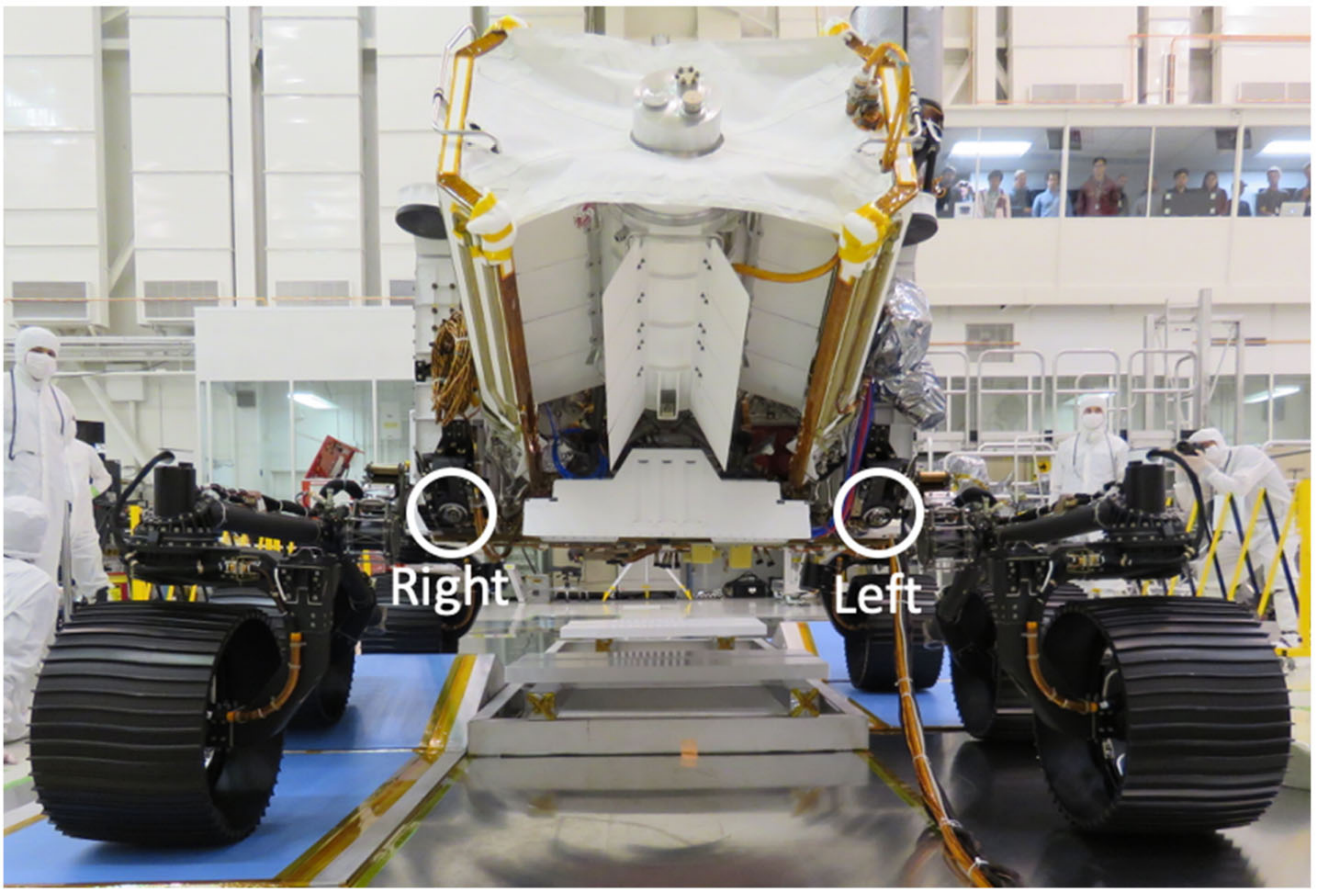
Perseverance Rear Hazcams

There are two Hazcams mounted on the rear of the rover, one on each side of MMRTG (Multi-mission Radioisotope Thermoelectric Generator). The stereo baseline between the two Rear Hazcam cameras is 93.4 cm. Unlike the Front Hazcams, the Rear Hazcams are connected to both RCEs. The Rear Hazcams are pointed $45^{\circ}$ below the nominal horizon. Each of the Rear Hazcams has a deployable cover assembly similar in design to the Front Hazcams. The Rear Hazcam covers are opened shortly after touchdown on Mars in a similar fashion to the Front Hazcams. Unlike the Front Hazcams, the Rear Hazcams do not have sun visors. Figure 15 shows a picture of the Rear Hazcams mounted on the rover. The proximity of the Rear Hazcams to the RTG exposes these cameras to a slightly higher radiation dose than the Navcams and Front Hazcams. Over the course of the prime mission, the cumulative effects of radiation on the Rear Hazcam detectors will result in higher dark current levels. Additionally, waste heat from the RTG warms the Rear Hazcams by $10~^{\circ}\text{C}$ to $20~^{\circ}\text{C}$ relative to the Front Hazcams, further raising the Rear Hazcam dark current levels. Both of these effects are also seen with the *Curiosity* rover Rear Hazcams. As with the *Curiosity* Rear Hazcams, these effects are not expected to impact the useability of the *Perseverance* Rear Hazcam images in any appreciable way during the prime mission. As the dark current increases over time, operational considerations may be made to acquire Rear Hazcam images during the cooler times of the day to reduce thermal dark current effects.

#### Navcams

**Fig. 16:**
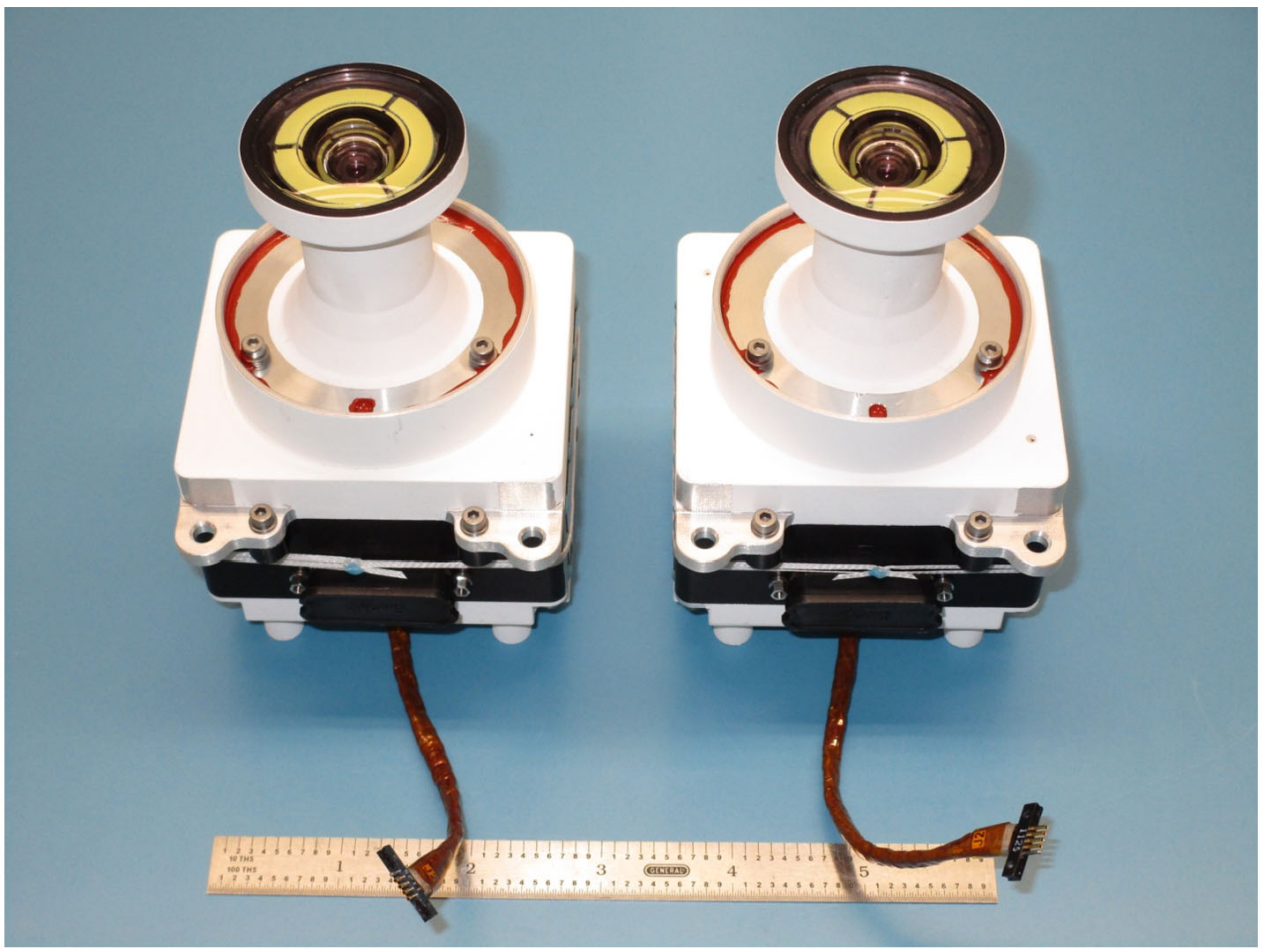
Flight Navcam cameras

**Fig. 17:**
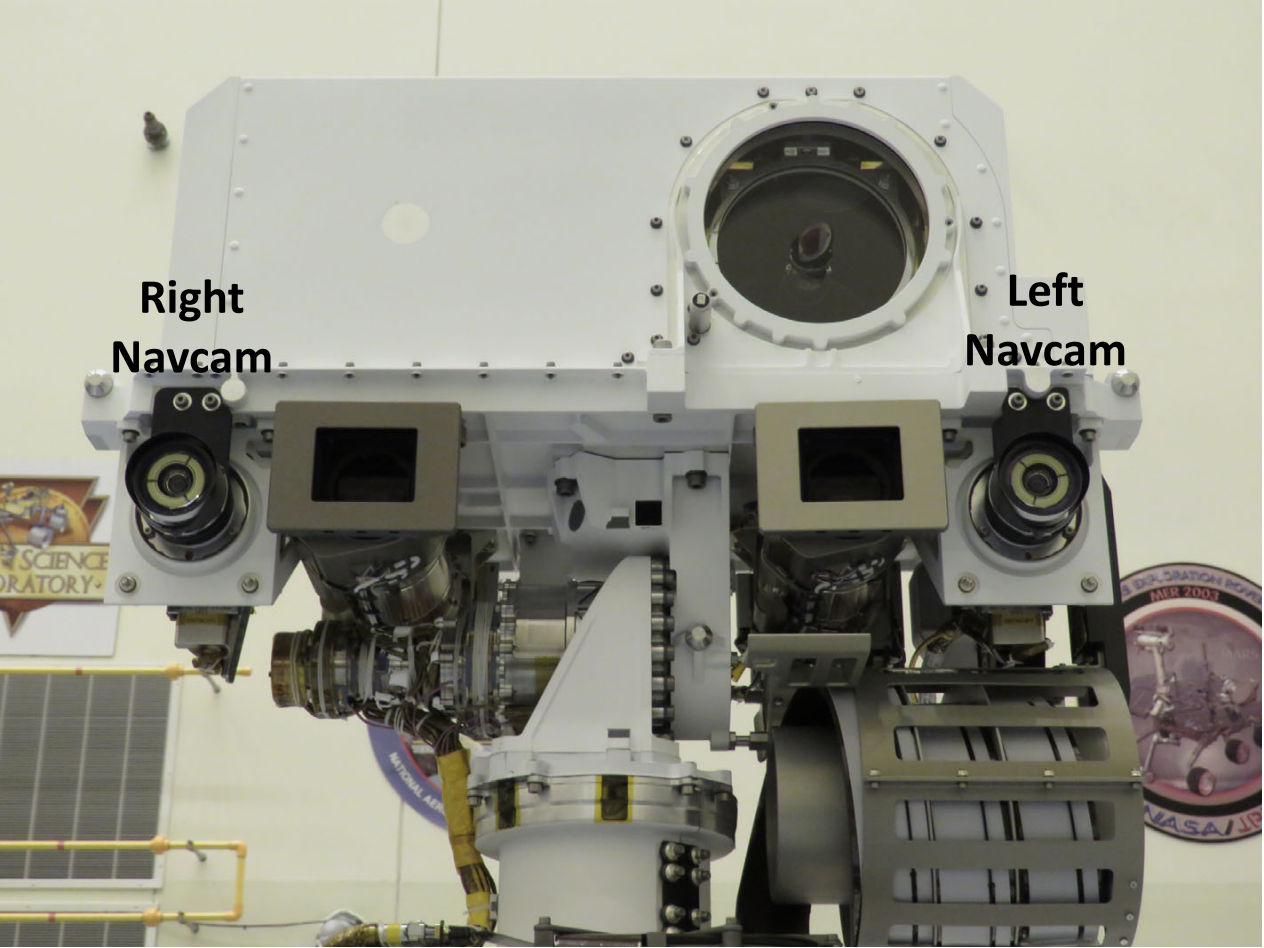
Closeup view of the Navcams mounted on the Remote Sensing Mast (RSM), a pan/tilt mast that points the cameras to targets of interest. The Navcams have a 42.4 cm stereo baseline. Also shown in the picture are the Mastcam-Z cameras (Bell et al. 2020, this issue) located between the Navcams, and the SuperCam (Wiens et al. 2020, this issue), located above the Navcams

The Navcam lenses have a focal ratio of f/12 and a best focus distance of approximately 3.5 meters. Each lens assembly contains six individual lens elements and a fused silica UV and IR blocking filter mounted between the powered elements and the detector. The Navcam field of view is $96^{\circ}$ (horizontal) by $73^{\circ}$ (vertical), with a diagonal FOV of $120^{\circ}$. The Navcam cameras are shown in Fig. 16. The Navcams are mounted to the underside of the camera plate on the Remote Sensing Mast (RSM) (Fig. 17).

#### Cachecam

The Cachecam has a fixed focus, 0.51 magnification lens with a depth of field of $\pm5~\text{mm}$ and a pixel scale of 12.5 microns/pixel. The Cachecam FOV forms a 50 mm diameter circle at the plane of focus. The Cachecam lens contains six individual lens elements. The Cachecam has no IR blocking filter because the LED illuminator has no output in the IR. At the end of the lens is an integrated illuminator assembly, which contains a mirror that redirects incoming light at an angle of $90^{\circ}$. The front of the illuminator is sealed with a fused silica window. The illuminator has a set of three light emitting diodes (LEDs) which allow the sample to be illuminated during imaging. The LEDs are powered whenever camera power is applied. The LEDs are made by Luxeon, part number LXZ1-4070, are white light LEDs with a CCT (correlated color temperature) of 4000 K.

**Fig. 18:**
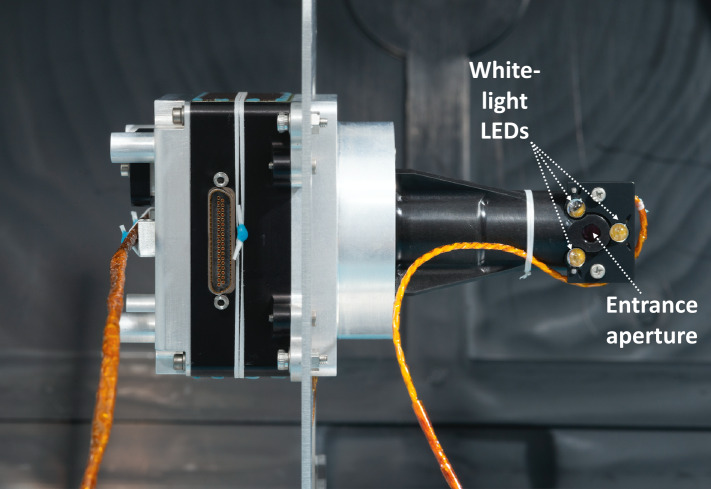
Flight Cachecam camera assembly, including the lens, illuminator, and camera body. This photo shows the view looking directly into the Cachecam entrance aperture

**Fig. 19:**
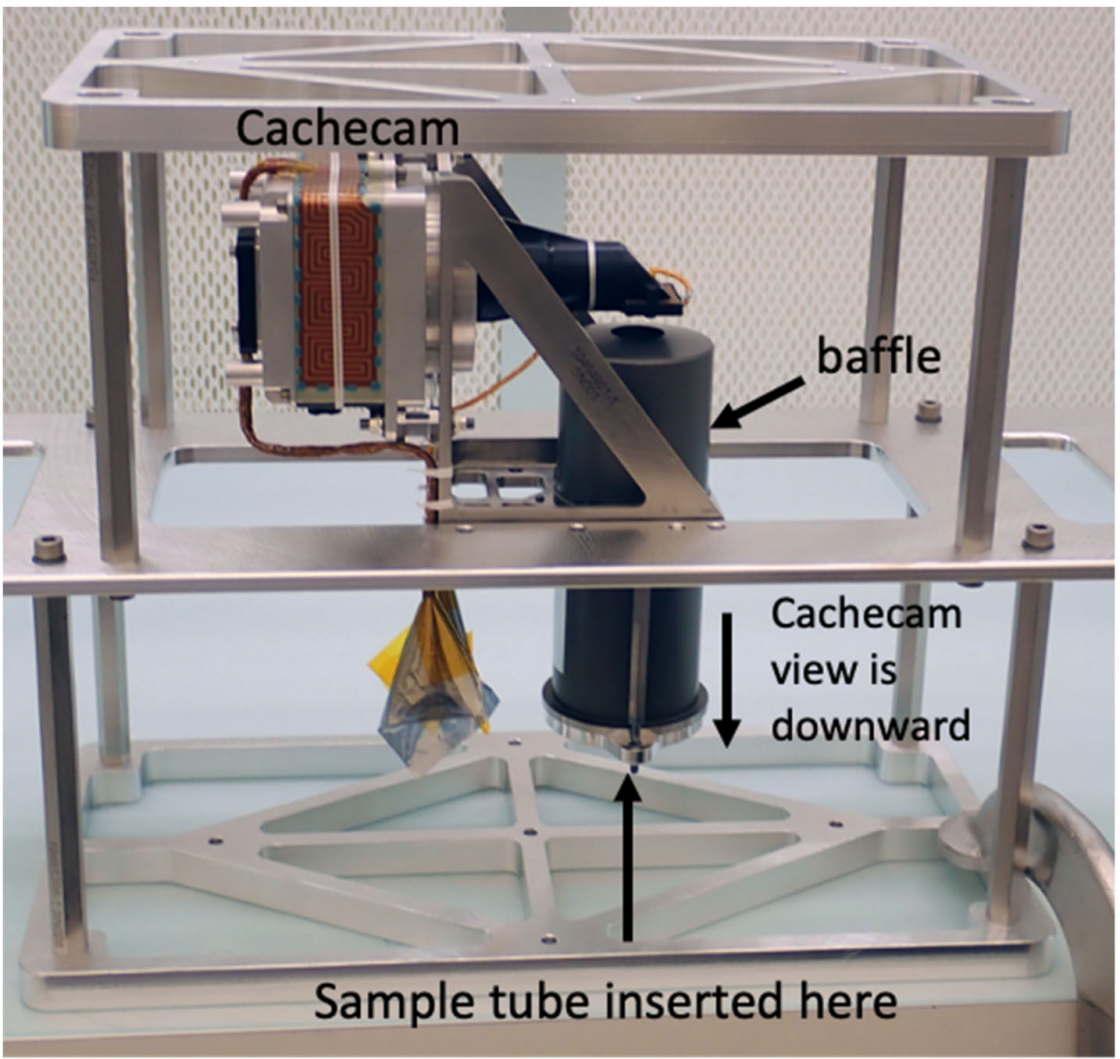
The *Perseverance* Vision Assessment Station, which includes the Cachecam camera and cylindrical baffle. Sample tubes are presented to the camera by a Sample Handling Assembly (SHA), a small robot arm that brings sample tubes into the baffle from the bottom (the SHA is not shown in this picture). The illuminator assembly contains 3 LEDs that shine down onto the sample tube from the top. The camera looks down into the tube and acquires images of the top of the material within the tube

**Fig. 20:**
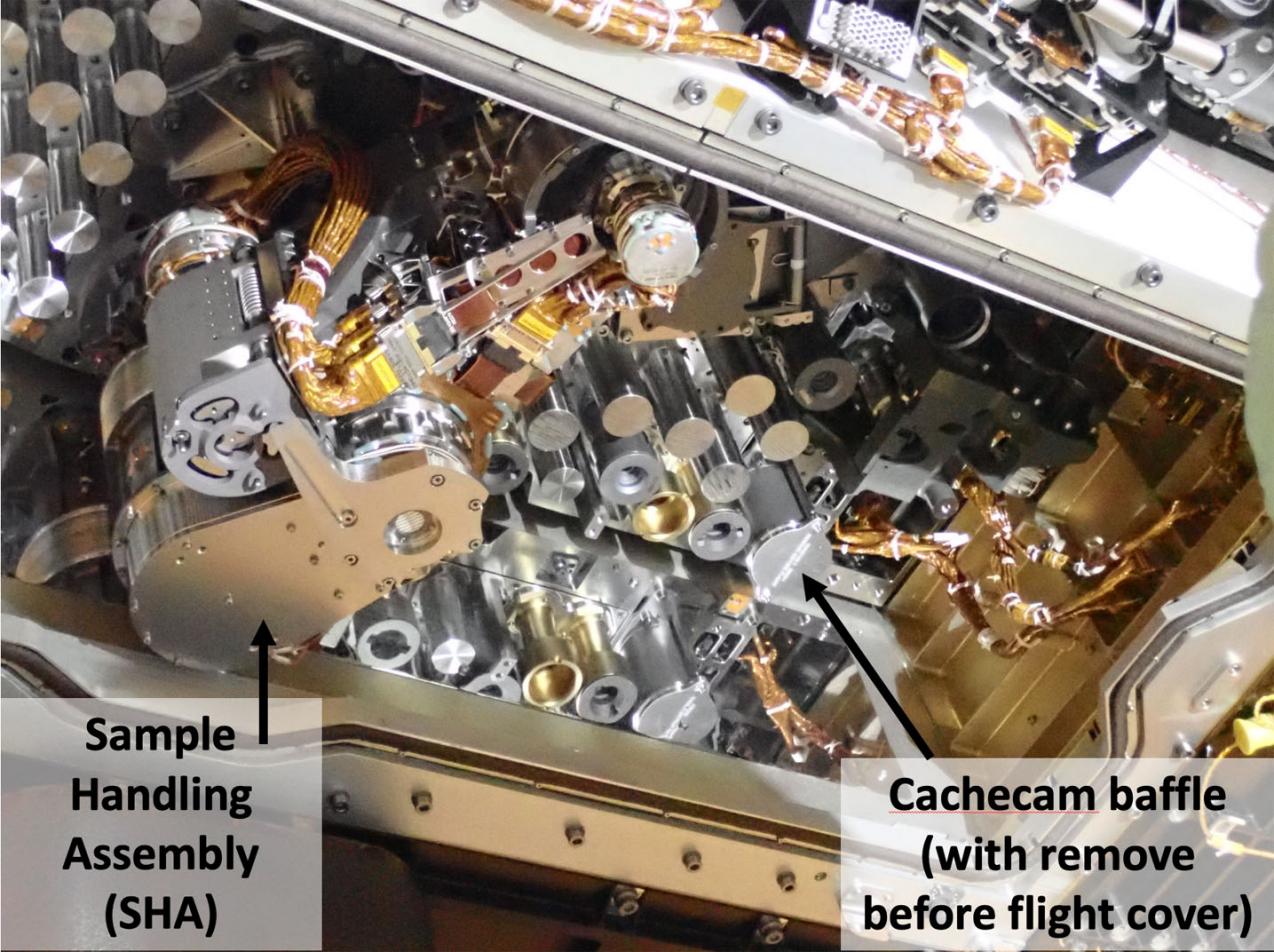
Location of the Cachecam within the Adaptive Caching Assembly (ACA), looking upwards from below the rover chassis. A portion of the Front Hazcam cover mechanism spring assembly can be seen in the upper right of the image

Figure 18 shows the flight Cachecam camera. The Cachecam is integrated into a sub-assembly called the Vision Assessment Station (Fig. 19). The Vision Assessment Station (VAS) contains a cylindrical shaped baffle sub-assembly. The VAS is placed into a larger assembly, the Adaptive Cache Assembly (ACA), which is inside the rover body (Fig. 20).

Due to the small depth of field of the Cachecam, the sample tube must be moved in and out of the plane of focus of the camera in small mm-sized vertical steps, with an image acquisition occurring at each step. The small depth of field was chosen to help estimate the height of the sample in the sample tube. The movement of the tube is done with the Sample Handling Assembly (SHA) robot arm. The SHA robot arm brings the tube over to the Vision Assessment Station and moves the sample tube through focus. The resulting set images form a Z-stack data set. The individual images are downlinked to Earth and processed. Information about the sample is extracted from this data set, including sample depth within the tube, sample texture, and estimates of the sample volume.

#### Camera Hardware Processing

##### ECAM Readout Modes

The MER/MSL camera data interface supports an image size of $1024\times 1024~\text{pixels}$, and the available image buffer sizes in the RCE RAM are limited to the MER/MSL heritage sizes. In order to use the heritage interface and memory allocation, a 20 Megapixel *Perseverance* ECAM image must be sent to the rover as a series of smaller sub-images (tiles). After an ECAM camera acquires an image, it stores the entire 20 Megapixel image temporarily in camera memory and returns individual, smaller sub-images to the rover upon command. The individual sub-image tiles are nominally $1280\times960$ pixels in size, and a total of 16 tiles must be transferred to copy an entire $5120\times3840$ pixel image into the rover computer. Smaller-sized sub-image tiles can be requested if desired. Larger tiles can also be requested, but they cannot be larger than $1296\times976$ pixels due to memory limitations in the RCE. The tile starting location can be arbitrarily placed within an image as long as the entire tile fits within the boundaries of the larger image and the starting locations are even multiples of 8.

The *Perseverance* cameras are also capable of reducing the pixel scale of an image prior to transmission by performing a pixel summing operation. There are four possible pixel scales available: full-scale ($1\times1$, or no spatial downsampling), half-scale ($2\times2$ spatial downsampling), quarter-scale ($4\times4$ spatial downsampling), and one-eighth scale ($8\times8$ spatial downsampling). In all modes the resulting subimage tiles are nominally $1280\times960$ pixels, with the exception of the $8\times8$ downsampling mode, which always produces $640\times480$ pixel images.

**Fig. 21:**
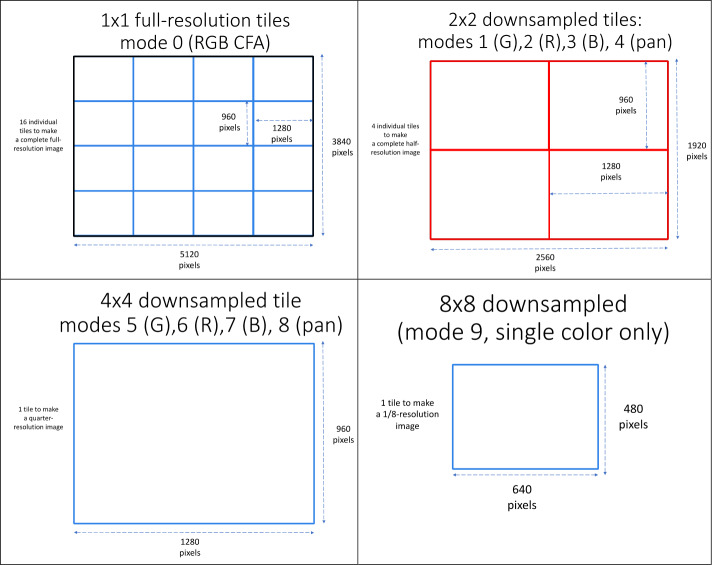
Schematic representation of the 10 available ECAM readout modes: **a**) full-scale ($1\times1$, upper left), **b**) half-scale ($2\times2$, upper right), **c**) quarter-scale (lower left), and **d**) 1/8th scale (lower right). In modes 0 through 8, all image tiles returned from the cameras are nominally $1280\times960$ pixels in size. In mode 9 the image tiles are $640\times480$ pixels in size. In the above figure the $1\times1$ and $2\times2$ tiles are shown aligned on even multiples of $1280\times 960$ for simplicity. In actuality the location of tiles can be located anywhere on the sensor as long as the entire tile is inside the larger source image and the starting locations are even multiples of 8

In addition to spatial downsampling modes, the *Perseverance* ECAMs also allow the separate readout of individual red, green, and blue color channels. Depending on the requested mode, the camera either subsamples the Bayer cells by sending only the requested color, or it averages all of the pixels of the same color together. The camera can also average all of the pixels together and return a panchromatic image. In the $8\times8$ mode, the camera always returns a panchromatic image by averaging all 64 pixels together into a single pixel. Table 3 lists all 10 available hardware processing modes, and Fig. 21 depicts the modes schematically.

**Table 3:** The 10 available color/pixel scale readout modes for the Perseverance ECAMs

Mode	Sub-image pixel scale	Sub-image tile size (pixels)	Description
0	1 × 1	1280 × 960	RGB, Raw Bayer pattern
1	2 × 2	1280 × 960	2 Green Pixel Average ($\frac{\sum G}{2}$)
2	2 × 2	1280 × 960	Red Pixel Subsample
3	2 × 2	1280 × 960	Blue Pixel Subsample
4	2 × 2	1280 × 960	4 Pixel RGB Average ($\frac{\sum R, G, B}{4}$)
5	4 × 4	1280 × 960	8 Green Pixel Average ($\frac{\sum G}{8}$)
6	4 × 4	1280 × 960	4 Red Pixel Average ($\frac{\sum R}{4}$)
7	4 × 4	1280 × 960	4 Blue Pixel Average ($\frac{\sum B}{4}$)
8	4 × 4	1280 × 960	16 Pixel RGB Average ($\frac{\sum R, G, B}{16}$)
9	8 × 8	640 × 480	64 Pixel RGB Average ($\frac{\sum R, G, B}{64}$)

##### Image Co-adding

The ECAM hardware allows in-camera co-adding of 2, 4, 8, or 16 images to improve the signal to noise ratio of the image. This capability may be useful when imaging shadowed surfaces and acquiring images under low-light conditions such as nighttime imaging of the surface, nighttime atmospheric imaging, and astronomy observations. During a co-add operation, the camera exposes an image, adds it to an image accumulator buffer, acquires the next image, and continues until the desired number of images have been co-added. In all cases the camera returns the final co-added image and discards the intermediate images. During the transmission of the image data to the RCE, the camera divides the image by the number of co-added images by performing a bit-shift operation and returning the most significant 12-bits of the co-added pixel data.

### EDLCAMs

#### EDLCAM Cameras

**Fig. 22:**
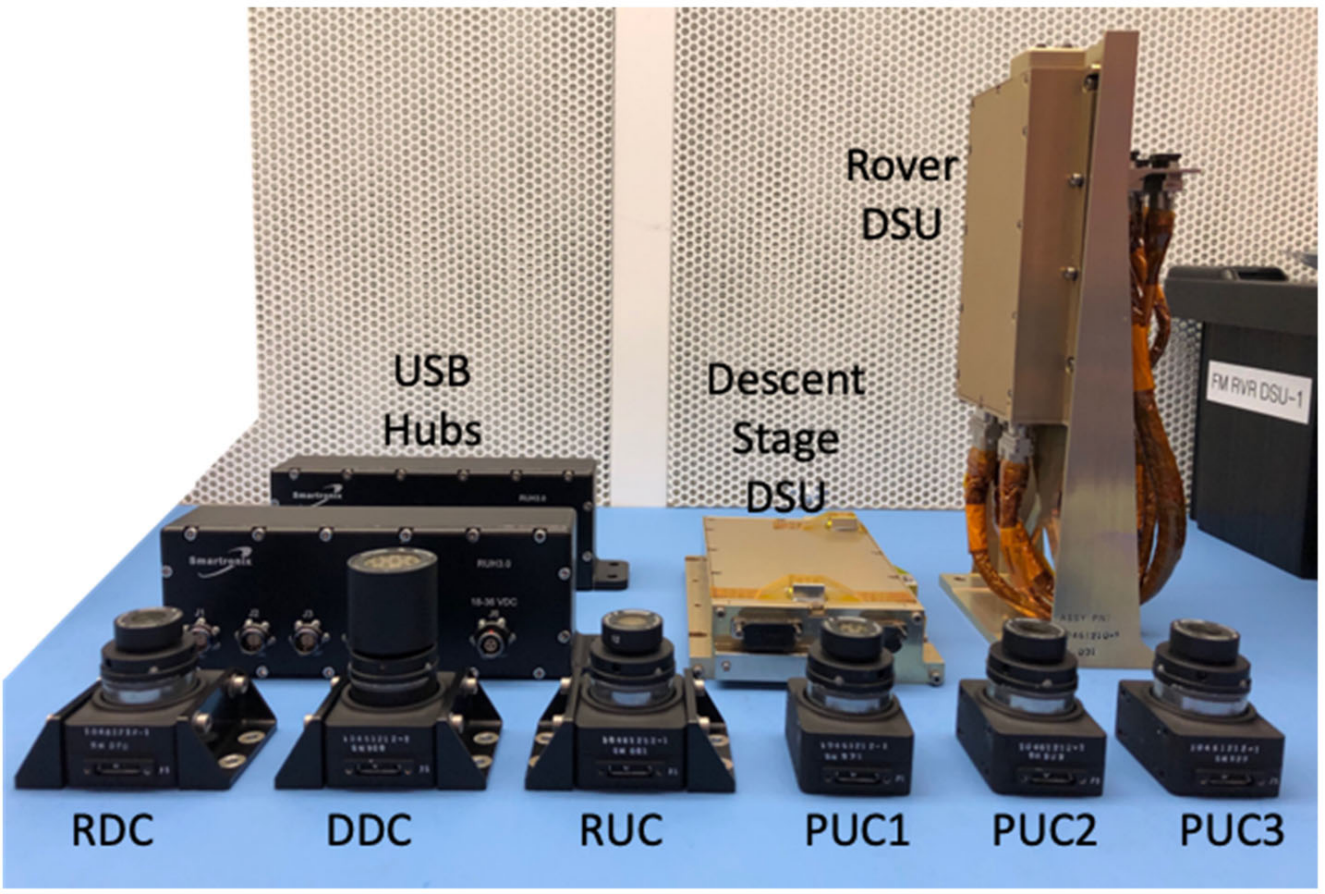
EDLCAM flight hardware

**Fig. 23:**
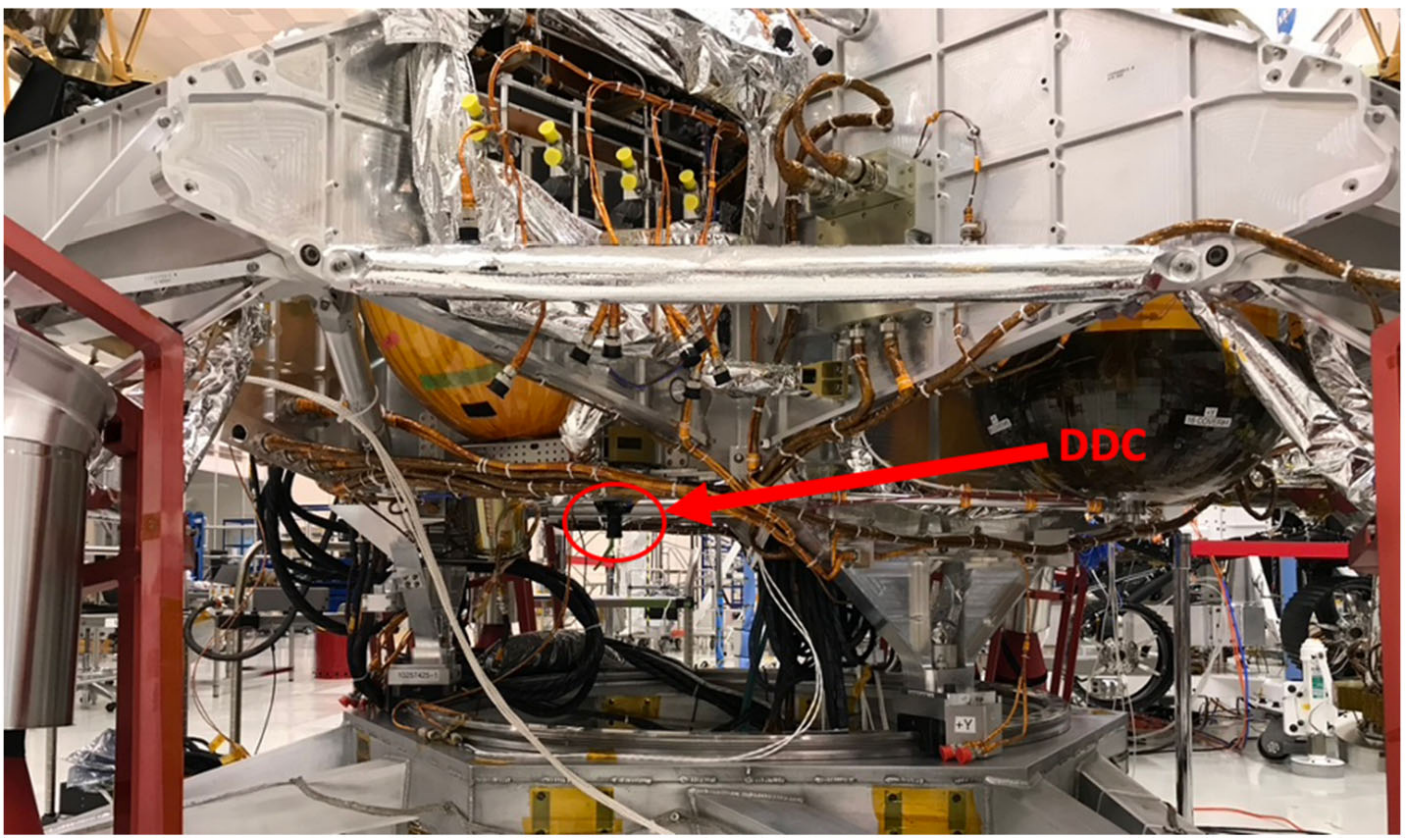
Location of the DDC on the descent stage

**Fig. 24:**
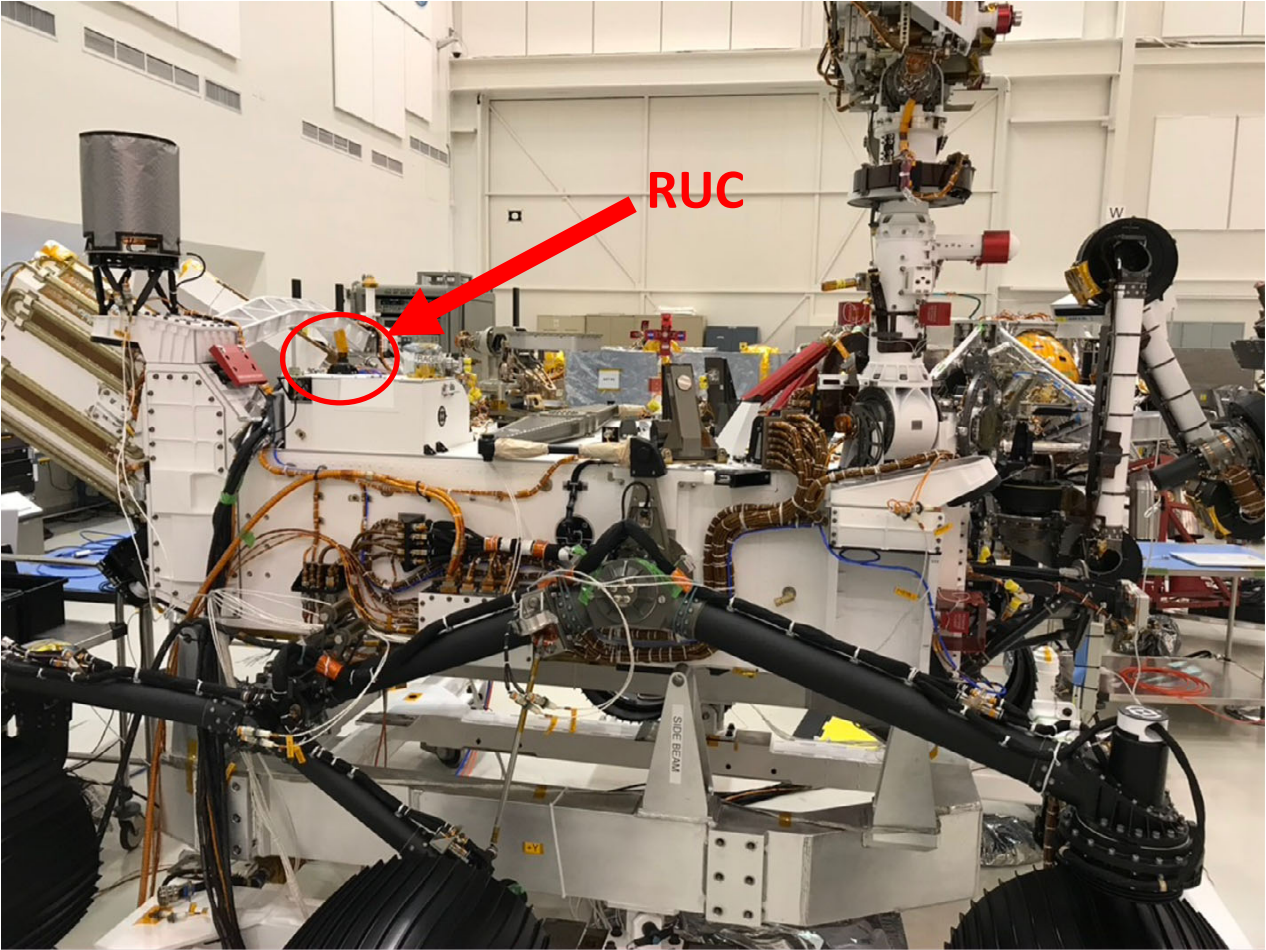
Location of the RUC on the rover

**Fig. 25:**
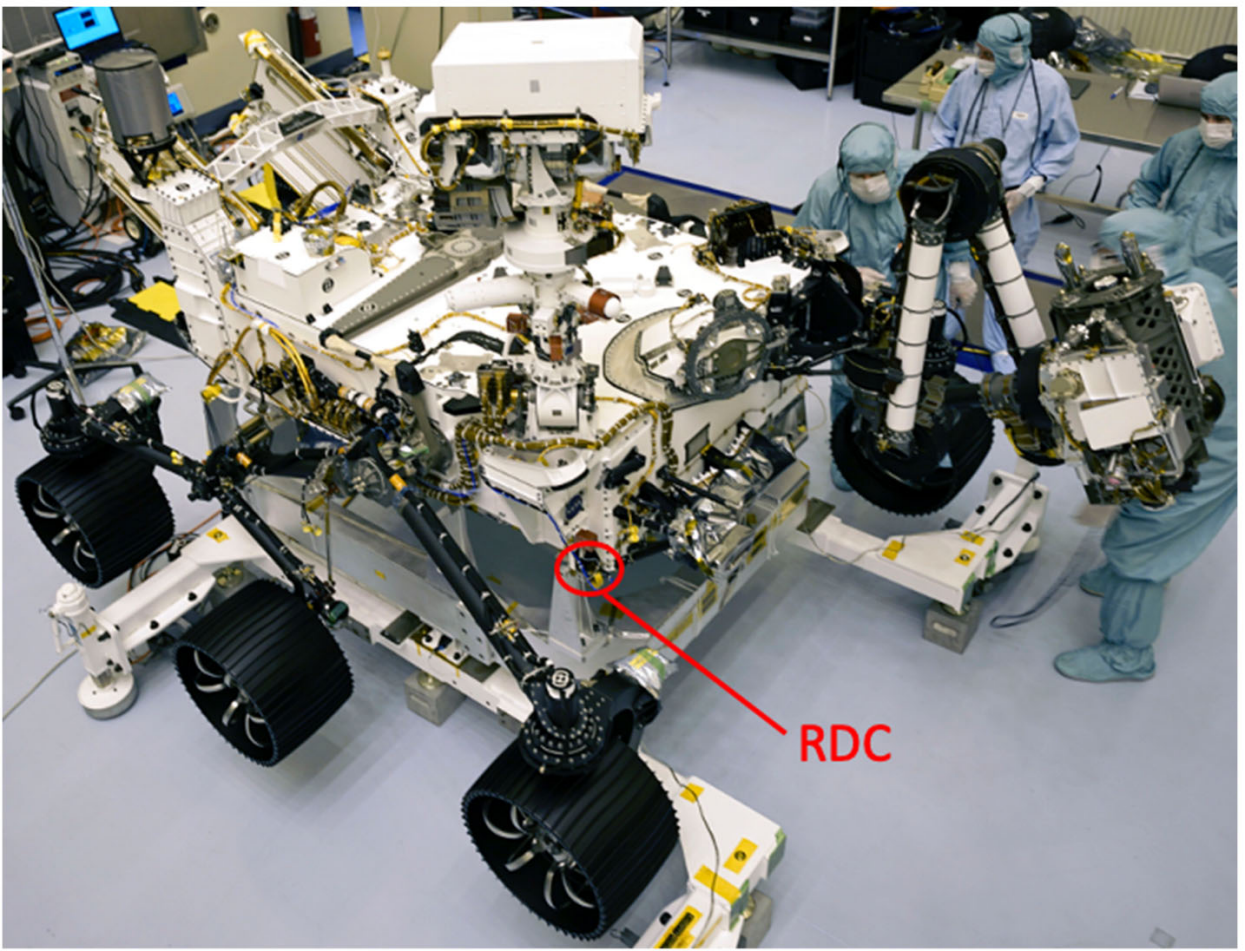
Location of the RDC on the rover

The EDLCAM camera bodies were manufactured by FLIR (formerly Point Grey). They have CS type lens mounts, which mate with custom lenses designed and manufactured by Universe Kogaku America. The PUC, RUC, and RDC lenses are identical in design and each contain 6 lens elements, including a front window, have a focal ratio of f/2.8, and a focal length of 9.5 mm. The DDC lens assembly contains 7 lens elements, including a front window. The DDC lens has a focal ratio of f/5.6 and has a focal length of 8 mm. See Table 4 for a summary of the EDLCAM camera types. The EDLCAM hardware is shown in Fig. 22, and the locations of the DDC, RUC, and RDC on the rover are shown in Figs. 23, 24 and 25.

**Table 4:** EDLCAM properties and estimated operating modes

Item	PUC (quantity 3)	RUC	RDC	DDC
Horizontal FOV	35^∘^ ± 3^∘^	35^∘^ ± 3^∘^	35^∘^ ± 3^∘^	48^∘^ ± 3^∘^
Vertical FOV	30^∘^ ± 3^∘^	30^∘^ ± 3^∘^	30^∘^ ± 3^∘^	37^∘^ ± 3^∘^
Pixel scale	$\sim0.5~\text{mrad/pixel}$	$\sim0.5~\text{mrad/pixel}$	$\sim0.5~\text{mrad/pixel}$	$\sim0.4~\text{mrad/pixel}$
Focal ratio	f/7	f/7	f/7	f/5.6
Focal length	9.5 mm	9.5 mm	9.5 mm	8 mm
Image Size (pixels)	1280 × 1024	1280 × 1024	1280 × 1024	2048 × 1536
Pixel Size	4.8 microns	4.8 microns	4.8 microns	3.45 microns
Camera type	CM3-U3-13Y3C-CS	CM3-U3-13Y3C-CS	CM3-U3-13Y3C-CS	CM3-U3-31S4C-CS
Detector	On Semi P1300 (RGB color)	On Semi P1300 (RGB color)	On Semi P1300 (RGB color)	Sony IMX265 (RGB color)
Mass (with lens)	80 grams	80 grams	80 grams	140 grams
Expected Frame Rate (and duration)	75 fps for parachute deployment ($\sim30~\text{seconds}$)	30 fps ($\sim 140~\text{seconds}$)	30 fps ($\sim 260~\text{seconds}$)	12 fps ($\sim75~\text{seconds}$)
	30 fps until backshell separation ($\sim98~\text{seconds}$)			
Estimated number of total images	5,190 per PUC (×3)	4,200	7,800	900
Estimated total number of images from all cameras	28,470			

#### Microphone

**Fig. 26:**
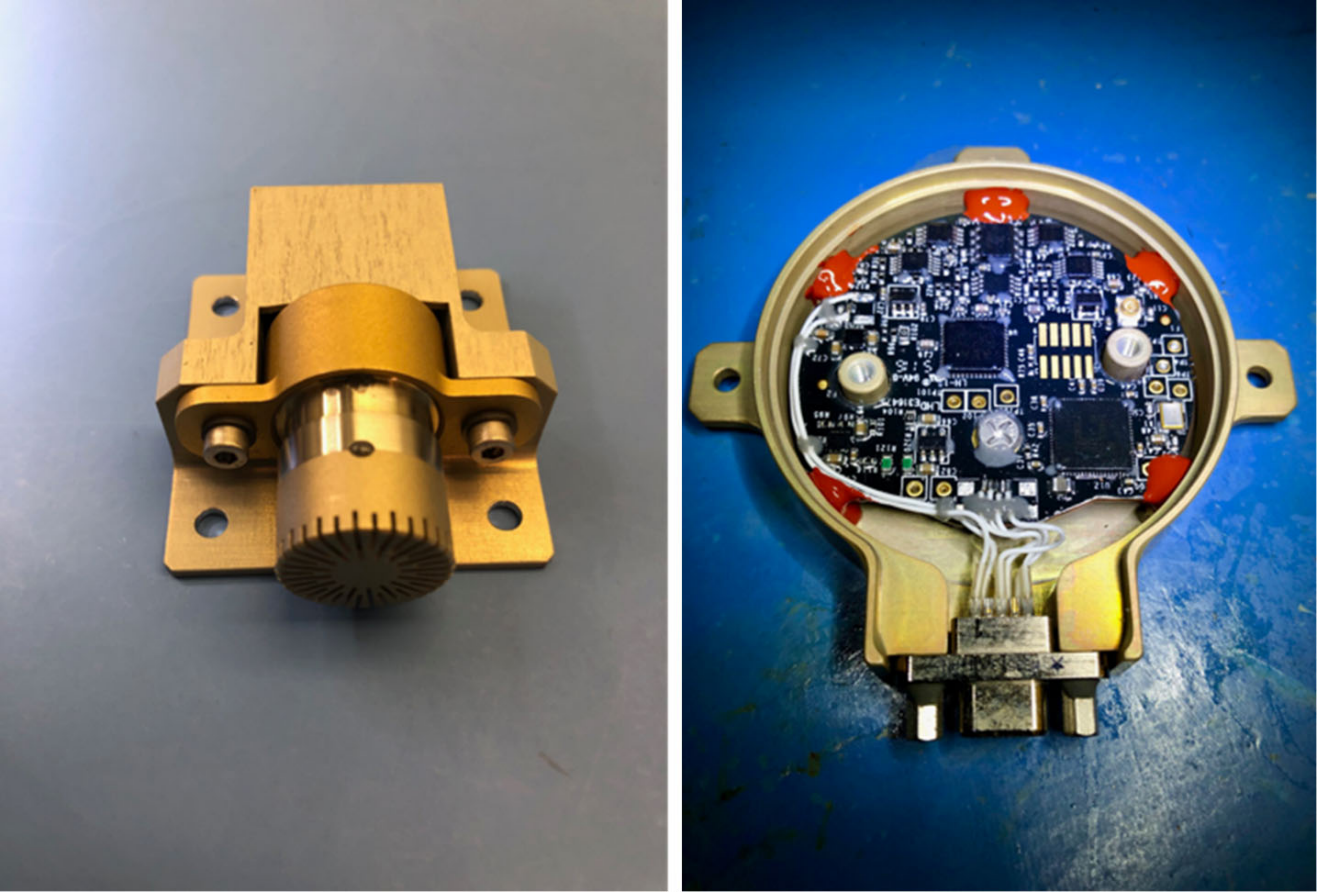
The EDLCAM microphone (left, with bracket) and digitizer assembly (right). The microphone is approximately $42~\text{mm}\times40 \times 19~\text{mm}$ and weighs 52 grams. The digitizer assembly is approximately 56 mm in diameter and weighs approximately 50 grams

**Fig. 27:**
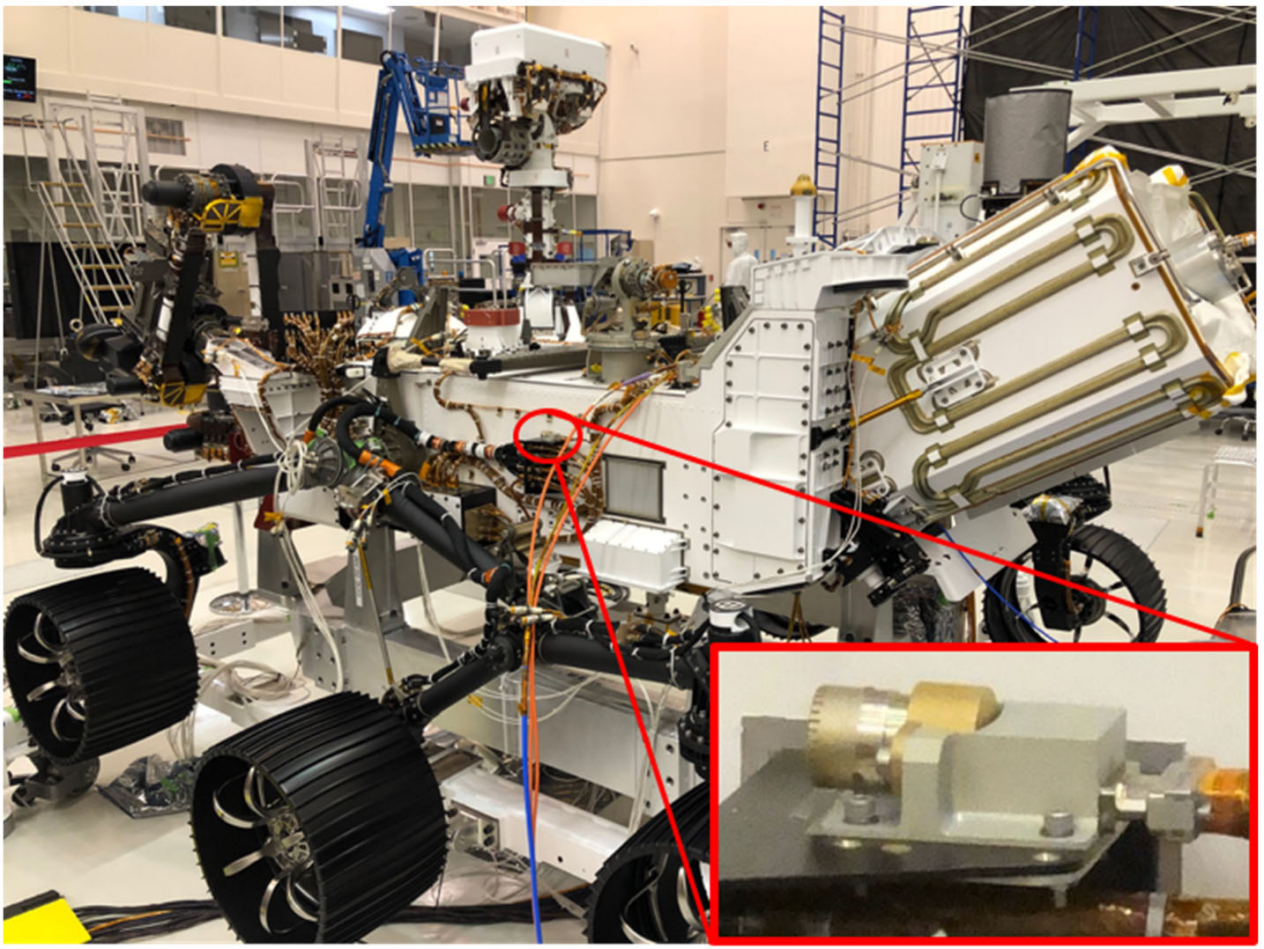
Location of the EDLCAM microphone on the *Perseverance* Rover. The microphone is located on the port side (Y-axis, rover coordinate frame) of the rover body, above the middle wheel

The EDLCAM system contains an omnidirectional microphone capsule for the capturing of sound during EDL. The microphone capsule was manufactured by DPA Microphones, part number MMC4006. The microphone capsule is connected to a DPA digitizer electronics board (part number MMA-A) that was repackaged into a custom aluminum chassis by the EDLCAM hardware development team. The digitizer board has two audio channels but the EDLCAM system only has one microphone capsule. The only key requirement on the microphone system was that it provided a simple interface to the EDLCAM DSU. The acoustic performance of the microphone system was not a key requirement – it has a frequency response from 20 Hz to 20 KHz ($\pm2~\text{dB}$). The digitizer is connected to the EDLCAM subsystem rover DSU via a USB2 connection. The microphone has a custom field grid that was modified for the Martian acoustic environment. The grid controls the behavior of sound waves on the diaphragm of the microphone while also minimizing the penetration of Martian dust into the diaphragm. Figure 26 shows the microphone capsule and microphone digitizer assembly. The microphone is mounted externally on the rover (Fig. 27).

Because the EDLCAM microphone is attached to the rover body, it will be commandable after landing. If the microphone survives the diurnal temperature cycles, it could be used to record Martian ambient sounds on the surface, mechanism movements such as wheel motions and coring operations, and other items of interest. The microphone could also be used in collaboration with the SuperCam microphone described in Murdoch et al. (2019), Chide et al. (2019), and Maurice et al. (2020).

#### Data Storage Unit (DSU)

In addition to six cameras and a microphone, the EDLCAM system includes two data storage units (DSUs) and two USB3 hubs. The DSU is an off-the-shelf computer-on-module (CoM) from CompuLab Ltd with an Intel Atom processor and solid-state memory. The DSU runs the Linux operating system, along with additional software to communicate with the EDLCAM sensors, perform the EDL data collection sequence, manage the data storage and compress the collected data files. The DSU uses a high-density connector to provide connectivity to the high-speed USB3, USB2, gigabit ethernet and SATA interfaces.

The main DSU is located inside the rover body. A second DSU, the descent stage DSU, is located on the descent stage. In both DSUs the CoM is connected to a custom electronics board that provides connectivity for all the USB devices. The two DSUs are almost identical to each other and communicate with each other through a gigabit ethernet link. The rover DSU includes a 480 GB solid-state flash memory drive (SSD) for data storage, provides a gigabit Ethernet link between both DSUs, and implements the high-speed serial communication protocol to communicate to the rover computer.

**Fig. 28:**
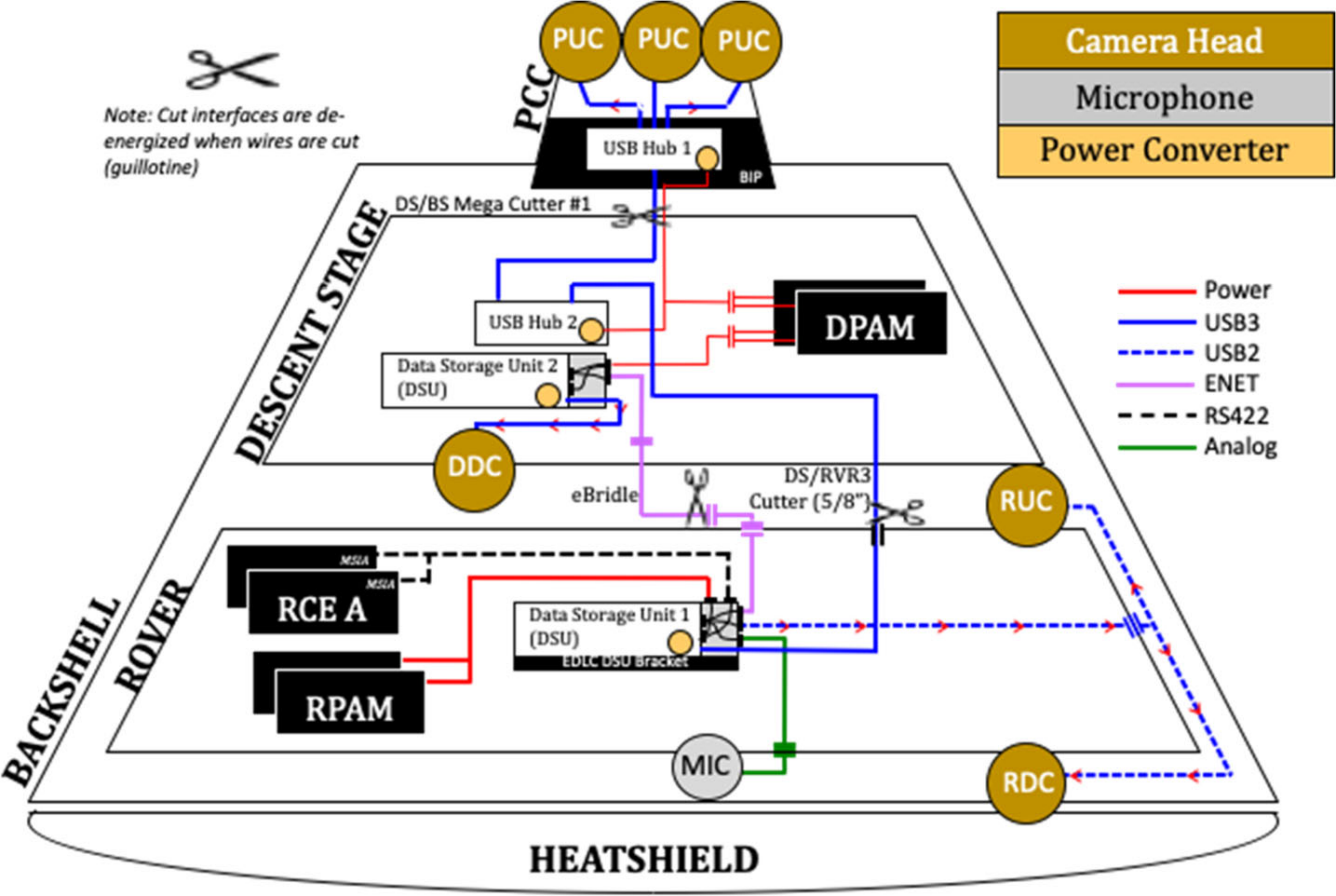
EDLCAM functional block diagram

The DDC streams data to the descent stage DSU over USB3, and the descent stage DSU streams data back to the rover DSU in real time over the ethernet link. The three Parachute Uplook Cameras (PUCs) connect to two USB3 hubs in series, which merge the USB3 stream into one port on the rover DSU and also acts as a USB repeater, allowing the data signals to travel beyond 5 meters. The RUC, RDC, and the microphone are USB2 devices and connect directly to the rover DSU. After the rover touches down on Mars, data saved on the rover DSU are available to be copied from the 480 GB NVM SSD into the RCE for subsequent downlink. Figure 28 shows a functional block diagram of the EDLCAM system.

#### Data Acquisition

A key feature of the design of the EDLCAM system is the requirement to not interfere with the safety of the vehicle during EDL. To meet this requirement the communication lines between the EDLCAM system and the rover are disabled at the hardware interface level during EDL to prevent spurious signals. The EDLCAM system runs autonomously once power is applied to the DSUs by the flight system. The application of power to the DSU and cameras is driven by external EDL events, as sensed by the flight system. Because the external triggering depends on the EDL system performance the exact number of images expected from each of the cameras is unknown in advance and can only be estimated. See Table 4 for the estimated number of images expected. The PUCs and DDC are jettisoned with the backshell and skycrane, but the RDC, RUC, and microphone remain on the rover and will continue to acquire data after touchdown.

##### PUCs

The three PUCs start to acquire image data immediately before parachute deployment at a frame rate of 75 fps (frames per second). After 30 seconds the frame rate drops to 30 fps until backshell separation, expected to occur approximately 98 seconds later. The total number of expected PUC images is $\sim5{,}190$ images per PUC, or 15,570 total images.

##### DDC

The DDC will start acquiring image data just before the rover separates from the descent stage and continues acquiring data through rover touchdown on the surface. The DDC acquires data at 12 fps for $\sim75$ seconds and is expected to acquire approximately 900 images.

##### RDC

The RDC will start acquiring data just before heatshield separation and will continue acquiring data through touchdown on the surface. The RDC acquires data at 30 fps for approximately 260 seconds and is expected to acquire approximately 7,800 images.

##### RUC

The RUC will start acquiring data just before the rover separates from the descent stage and continues acquiring data through rover touchdown on the surface. The RUC acquires data at 30 fps for approximately 140 seconds and is expected to generate approximately 4,200 images.

##### Microphone

The microphone will acquire data from immediately before parachute deployment through post-touchdown. The expected data acquisition duration is approximately 287 seconds. The microphone records at a sampling rate of 48 kHz, digitized at 24 bits.

### LCAM

The LCAM characteristics are listed in Table 5.

**Table 5:** LCAM characteristics

Horizontal FOV	90^∘^
Vertical FOV	90^∘^
Diagonal FOV	118^∘^
Focal ratio	f/2.7
Focal length	5.8 mm
Best focus	2 meters to infinity
Pixel scale	1.67 mrad (2 × 2 summed pixels)
Mass	880 grams
Volume	$82~\text{mm}\times 102~\text{mm}\times 154~\text{mm}$
Boresight mounting direction	-Z (pointed straight down)
Detector Type	On Semiconductor Python 5000
Pixel format	1024 × 1024 pixels (2 × 2 summed mode), windowed from a total format of 2592 × 2048 pixels
Pixel pitch	9.6 microns (2 × 2 summing of 4.8 micron pixels)
Optical format	$9.83~\text{mm}\times 9.83~\text{mm}$
Pixel type	global shutter with CDS
Full well	33,925 e- (effective with 2 × 2 summing, gain 1)
System noise	32.6 e-
Conversion gain	139.4 e-/DN (2 × 2 summing)
Maximum SNR	45 dB
Dynamic range	60 dB
Filter	480 nm to 720 nm
QE	55% peak
Command/data interface	LVDS/ChannelLink
Voltages	4.25 V to 5.5 V
Data rate	480 Mbps video output
Power	3.7 Watts
Memory	32 Gbytes flash
FPGA	MicroSemi Rad-tolerant (RTAX-SL)
Radiation tolerance	TID RDF >10
Temperature range	$-25~^{\circ}\text{C}$ to $+50~^{\circ}\text{C}$ (operational)
	$-40~^{\circ}\text{C}$ to $+70~^{\circ}\text{C}$ (survival)

**Fig. 29:**
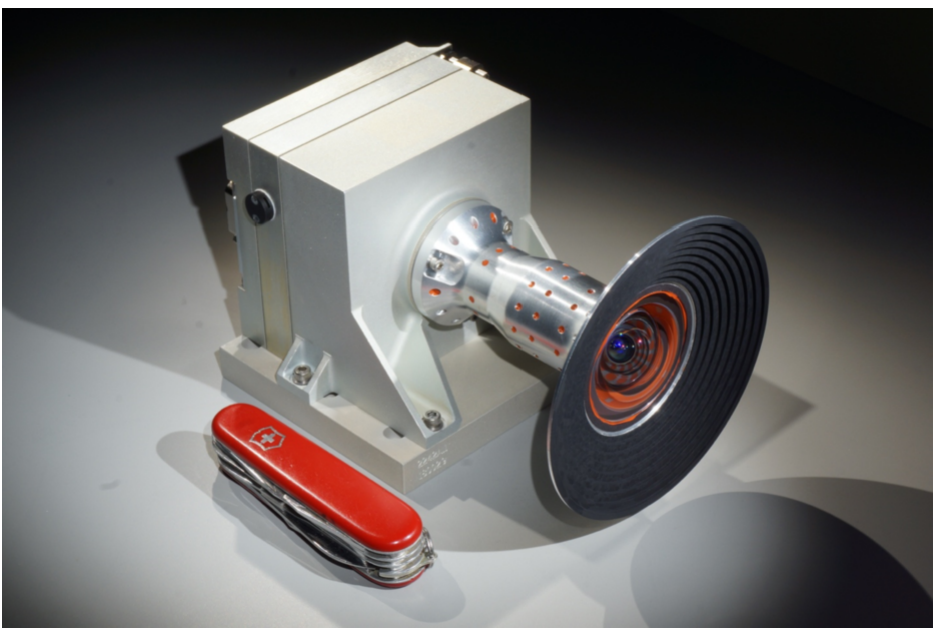
The LCAM flight unit, just prior to delivery to ATLO

**Fig. 30:**
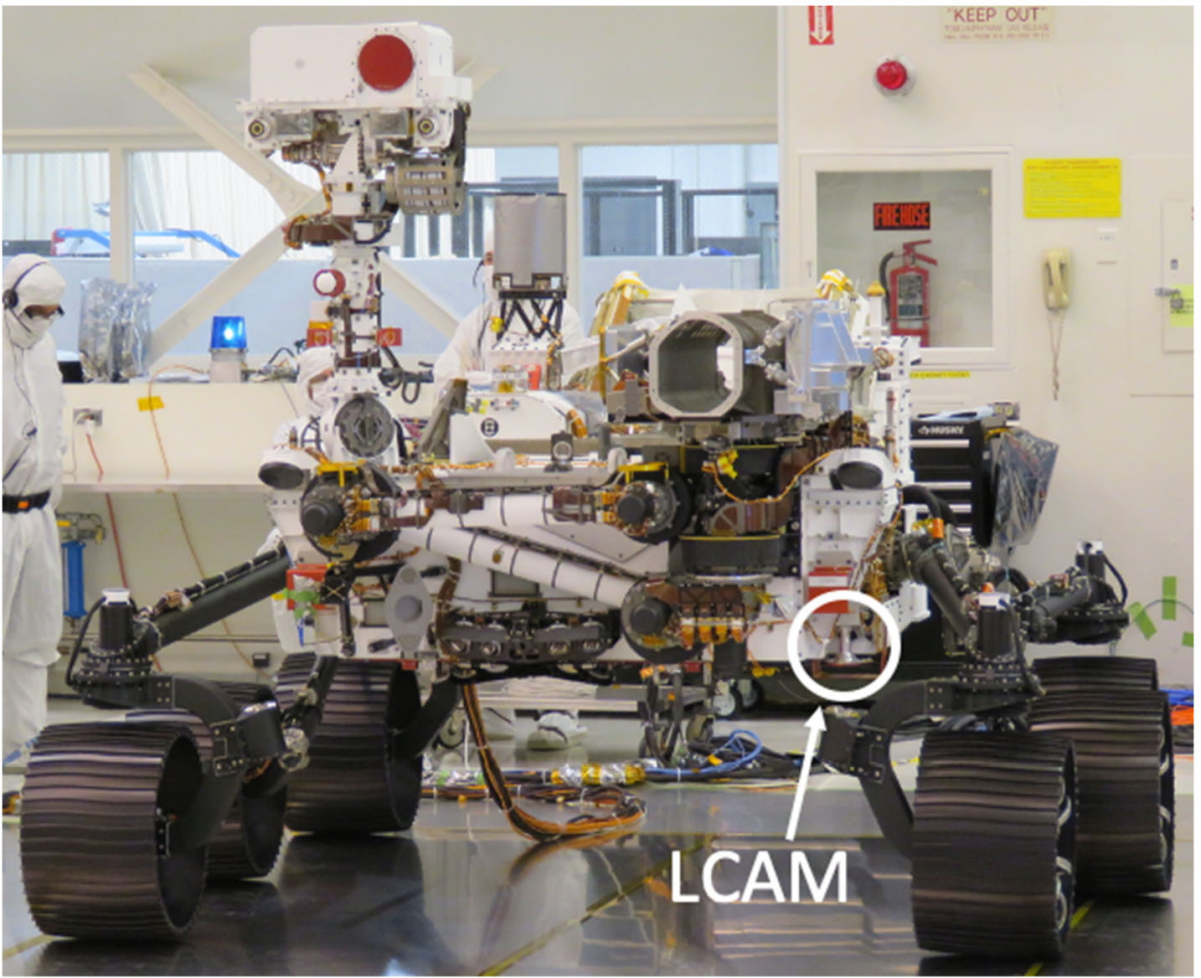
Location of the LCAM on the rover

An image of the flight LCAM is shown in Fig. 29. Figure 30 shows the LCAM mounting location on the Perseverance rover.

#### Optics

The LCAM lens is a 9-element, all-refractive lens with a nominal horizontal and vertical FOV of $90\times90$ and an effective focal length of 5.785 mm. The nominal on-axis f/# is 2.7. The LCAM lens was fabricated at Collins Aerospace with design support from Synopsys Optical Design Services.

#### Detector

The detector is an On Semiconductor Python 5000, a global-shutter CMOS image sensor with $2592\times2048\times4.8~\upmu \text{m}$ pixels and on-chip 8-bit or 10-bit digitization. LCAM uses the monochrome version of the sensor.

#### Electronics

The LCAM electronics design is derived from two previous camera designs by MSSS: the VSS (Vision Sensor System) camera on the NASA Goddard Space Flight Center (GSFC) Restore-L Mission and the P50 camera on the Naval Research Laboratory (NRL) Robotic Servicing of Geosynchronous Satellites (RSGS) Mission. Both of these cameras used the On Semiconductor Python 5000 detector. The LCAM electronics includes three printed circuit assemblies: the Interface Adaptor (IFA), Digital Module (DM), and Focal Plane Assembly (FPA).

The IFA connects to the LVS via a 25-pin Micro-D interface connector and communicates using asynchronous command and telemetry interface signals (LVDS), a discrete LVDS trigger, and a ChannelLink video interface. The DM contains a Microsemi RTAX FPGA and four NAND flash banks, which for LCAM are only used to store redundant copies of non-volatile operating parameters. The FPA contains three Line Current Limiter (LCL) modules that control the three power supplies to the sensor.

#### LVS Interface

In response to trigger signals, LCAM acquires each image, optionally sums it $2\times2$, and transmits it to the Vision Compute Element (VCE) component of the LVS. Images are always summed $2\times2$ and sent with 8-bit pixel depth. The ChannelLink clock is run at 35 MHz by default, with an optional mode at 17.5 MHz. Two pixels are sent in each ChannelLink cycle.

## Flight and Ground Software

### ECAM Flight Software

The ECAM flight software runs in the RCE. The software is a copy of the MER ECAM IMG software module described in Maki et al. (2003), Litwin and Maki (2005), modified for MSL (Maki et al. 2012), and subsequently modified for Mars 2020. The flight software handles camera control, command handling, and the post-processing of camera images after they are transferred over to the RCE. The software is mostly identical to the MER/MSL versions, and because of this the reader is referred to the above manuscripts for a more detailed description. Key changes for Mars 2020 are described here.

#### Image Acquisition and Readout

*Perseverance* ECAM images are exposed using either manual or auto exposure, using the methods described in Maki et al. (2003). On Mars 2020 the resulting full-scale image remains stored in camera memory until a new image is acquired or the camera is powered off. As described in Sect. 3.1.6, the *Perseverance* engineering cameras do not send back the entire $5120\times 3840$ pixel image in a single image transfer due to limitations available RAM for image buffers. Instead, image data are sent back in nominally-sized $1280\times 960$ pixel sub-images (tiles). In order to read out a full-scale image, 16 individual image acquisition commands must be sent to the camera, with each command resulting in the transfer of a single $1280\times 960$ pixel image tile to the RCE. The 16 individual image tiles are saved as 16 separate files and sent back to Earth. The individual image files are reassembled back into the original full-scale image using ground software.

The image tiling architecture imposes a requirement that the image acquisition commands must distinguish the difference between exposing the sensor and reading out a previously-exposed image from camera memory. The Mars 2020 ECAM flight software accommodates this difference by repurposing the “NONE” exposure type from MER and MSL. On the MER/MSL rovers, an exposure type of NONE would power on the cameras and prepare for acquisition, perform any necessary pan/tilt pointing if applicable, but not acquire an image. On *Perseverance*, the NONE exposure type is used to perform the transfer of an image tile from a previously-exposed image. The flight software supports two modes of camera hardware operation: 1) acquire an image and immediately transmit a tile, and 2) transmit a tile from the current image in camera memory without re-exposing the sensor.

Full-scale image acquisition with the Mars 2020 IMG FSW will typically be performed in the following way: the first IMG_ACQUIRE command will request an exposure type of AUTO, in camera mode 6, which corresponds to a $4\times4$ downsampled, full FOV, red channel image. This will expose the sensor to the scene of interest using autoexposure over the full field of view. The exposure command would be followed by 16 subsequent IMG_ACQUIRE commands, each requesting an exposure type of NONE (readout only) in mode 0, which would transmit a full-scale image tile ($1\times1$ downsampling, Raw Bayer) from camera memory into the RCE.

Alternatively, a user may require that the 16 image tiles be autoexposed individually. This would be performed by requesting 16 separate autoexposure commands, each with different tile coordinates. The FSW allows any of the 10 available camera hardware modes to be used for tile readout. Autoexposure can be run using any of the 10 available readout modes. This enables the autoexposure of an image based on a single color channel in the $2\times2$ and $4\times4$ modes. Images can also be autoexposed in all color channels individually. Alternatively, a single panchromatic autoexposure could be used to set the exposure time for the image, with a subsequent readout of the red, green, and blue channels. A detailed discussion of exposure strategies is beyond the scope of this manuscript, but it is expected that different use cases will employ different exposure and readout strategies.

#### Image Compression

In addition to the ICER wavelet and LOCO compression used in Maki et al. (2003) and described by Klimesh et al. (2001) and Kiely and Klimesh (2003), the *Perseverance* IMG module inherits a lossy JPEG (Joint Photographic Experts Group) compressor from the Mars InSight lander imaging system (Maki et al. 2018). ICER and LOCO support 8-bit or 12-bit pixels. The JPEG compressor supports both greyscale and color image compression, selectable compression quality, and selectable chroma subsampling for color images. The JPEG code supports 8-bit pixels only. The JPEG code is originally from the Independent JPEG Group (http://ijg.org) modified by InSight to run in the VxWorks environment. Mars 2020 inherited the JPEG code from InSight.

Color images are typically compressed using color JPEG compression, although the FSW allows the individual color channels to be compressed separately if desired. Compressing individual color channels separately is done via greyscale compression (ICER or JPEG) on each of the individual channels, and incurs a significant ($\sim3\times$) data volume penalty over JPEG color compression. JPEG color compression is much more efficient: most of the compressed data volume of a color JPEG image (typically 90% or more) is the luminance data, with the color data comprising 10% or less of the total volume. In some cases, when a high-quality, full-scale image is desired, the raw Bayer pattern can be compressed using LOCO lossless compression. The FSW allows many combinations of acquisition and compression to cover the various use cases during surface operations. Demosaicking of raw Bayer tiles is done using the algorithm by Malvar et al. (2004), using software code inherited from the Mars InSight lander imaging system (Maki et al. 2018).

### EDLCAM FSW

All EDL camera images and sound files are saved as raw data in the DSU. The raw data can be compressed and saved separately in the DSU as compressed files. Either compressed or raw data can be copied into the rover. Data can be compressed multiple times if desired and saved to separate files for later transmission. Most video data will be compressed using MPEG (Moving Picture Experts Group) video compression. Individual images will generally be compressed using lossy JPEG compression.

The EDLCAM DSU runs a custom-built version of Linux that has been tailored to maximize data throughput from the USB cameras to the rover DSU non-volatile storage (SSD). The application layer consists of two modules that determine the two operating modes: command mode and EDL mode. Upon power-on, the system waits for commands from the rover flight software. If no commands are received after a pre-determined timeout, the EDLCAM software transitions to EDL and begins autonomously collecting data from the cameras. Because the DSU does not receive any commands from the rover during EDL, the entire EDL sequence is autonomous. If the DSU receives a command before transitioning to EDL mode, the EDLCAM software transitions to command mode. While in command mode the DSU sits idle until additional incoming commands are received from the rover. During surface operations, commands will be sent to the DSU to initiate internal data compression and subsequent file transfer into the RCE.

The core EDL data processing and compression engine is powered by FFMPEG (ffmpeg.org). The JPL-developed custom software application layer is comprised of approximately 20,000 lines of code. All other functionality is provided by open-source software projects. The total software storage footprint is less than 100 MB. During the 7-minute EDL sequence, the EDLCAM system is expected to generate and store more than 40 GB of camera and microphone data.

### LCAM

LCAM has no software; all of the software for LVS is resident in the VCE. LCAM images are exposed using exposure times computed in advance based on predictions of the signal level under the expected illumination conditions. The VCE has the capability to dynamically change the exposure time should the images appear too bright or too dark.

### Ground Software

**Fig. 31:**
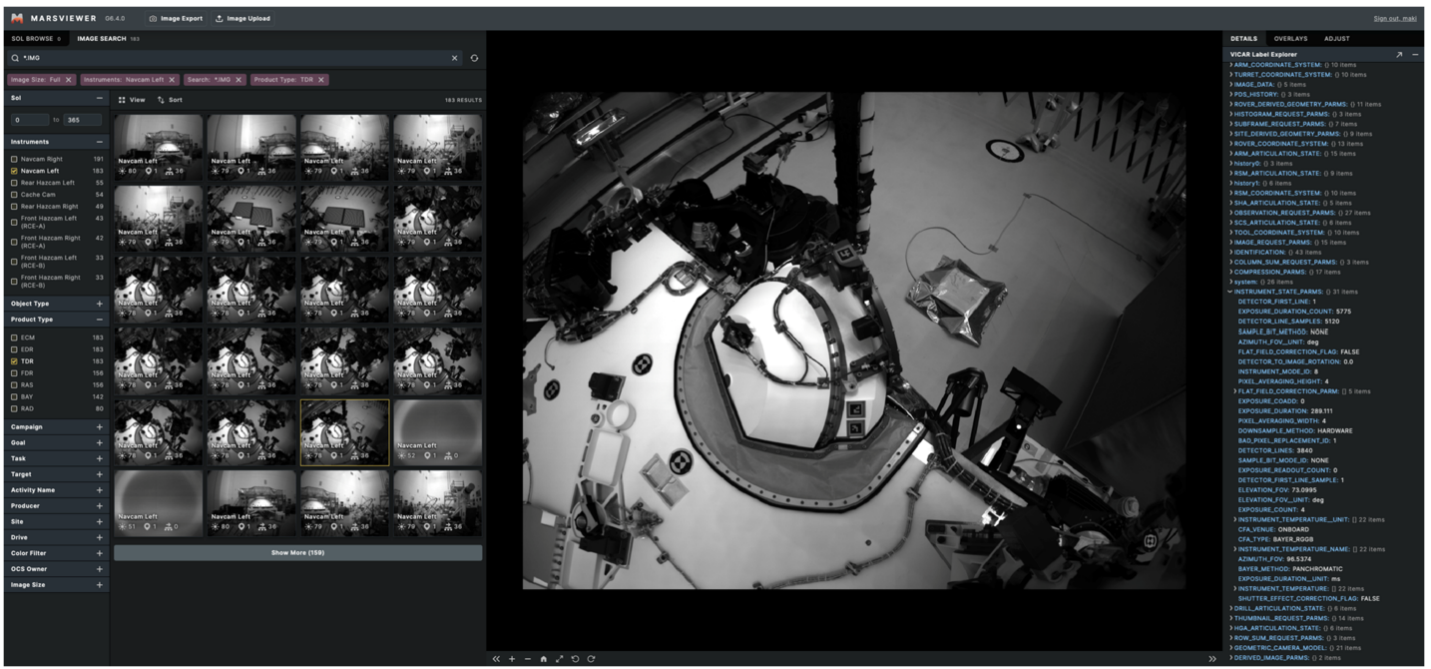
The Mars 2020 web-based image viewing and data search tool

The *Perseverance* ground image processing system is largely inherited from Mars Pathfinder (LaVoie et al. 1999) and MER (Alexander et al. 2006), with subsequent updates for MSL and the Mars InSight mission (Abarca et al. 2019). Many of the updates for Mars 2020 include adding capabilities specific to the handling and assembly of the individual image tiles into the larger original images. Another improvement to the heritage ground system involves the development of web browser-based image viewing and cataloging tools (Fig. 31).

## Calibration and Test

### ECAMs

**Fig. 32:**
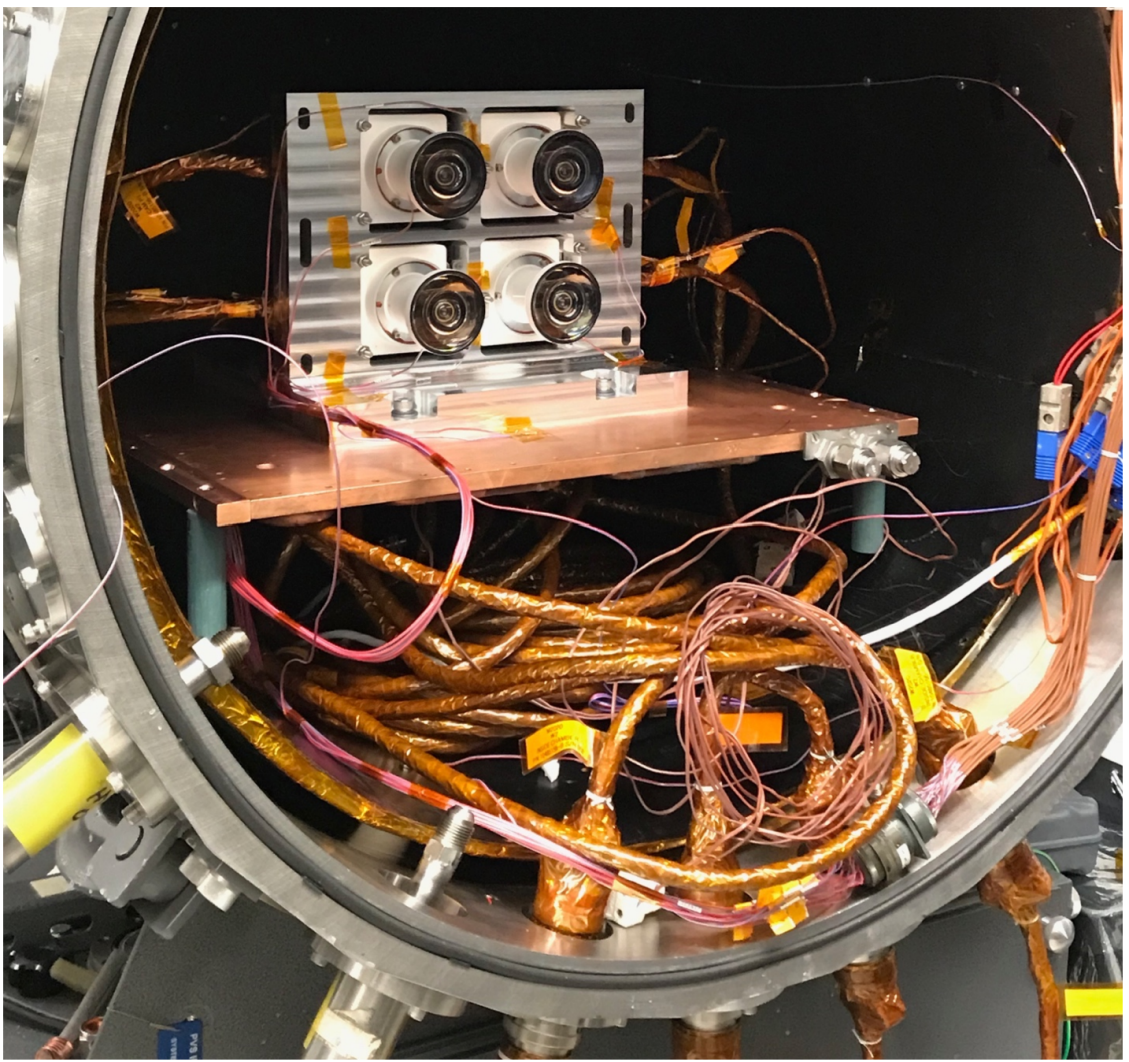
Instrument-level thermal vacuum testing of the *Perseverance* Hazcams

The Navcams, Hazcams, and Cachecam underwent instrument-level acceptance testing, functional testing, and calibration prior to delivery to ATLO (Fig. 32). Calibration of the ECAM cameras included the characterization of MTF (Modulation Transfer Function), SNR (signal to noise ratio), boresight location, FOV, flat field, dark current, and color response function.

#### Stereo Imaging Tests

**Fig. 33:**
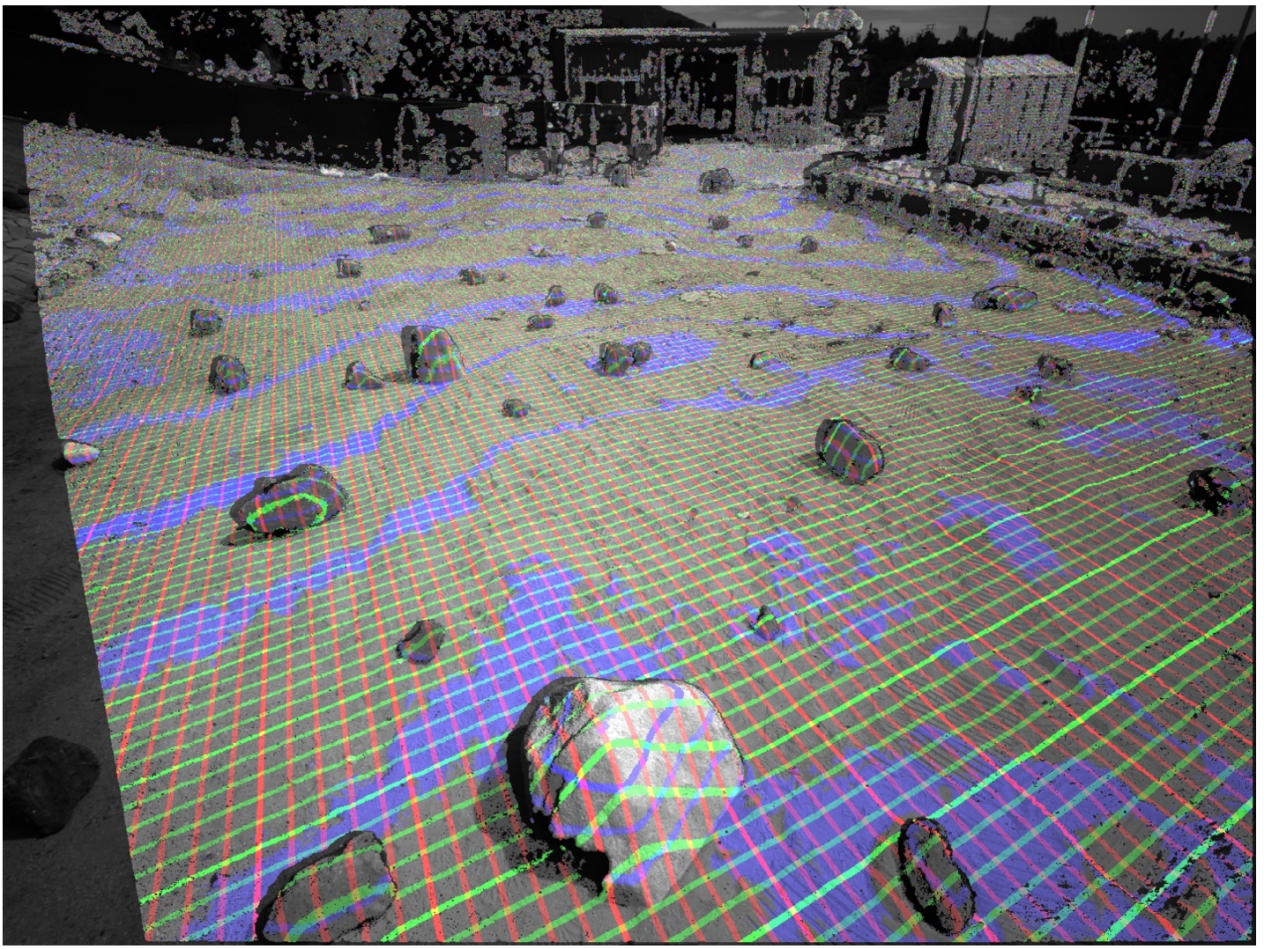
Results of stereo processing on a pair of 20 Megapixel prototype Navcam images in the JPL Marsyard. The higher pixel scale of the M2020 Navcams (compared to MSL) allows denser stereo maps at the same camera-to-object distance. The space between XYZ contours in the above figure is 10 cm. The red contour lines represent distance in the X direction of the local site coordinate frame and the green contours represent the distance in Y. The purple contours represent the distance in Z (height)

The Mars 2020 stereo image processing system has been tested with data from the flight unit cameras and flightlike prototype cameras. Because the *Perseverance* ECAMs are capable of generating images at four different pixel scales, a preprocessing system re-assembles the image tiles back into larger images (as applicable) prior to running the stereo matching software on the stereo pairs. The pre-processing system generates several versions of the reassembled images, resulting in a set of images with different pixel scales and use cases. A typical example is shown in Fig. 33. A full description of the image products will be included in the archive delivery to the NASA Planetary Data System (PDS).

#### Solar Imaging Tests

**Fig. 34:**
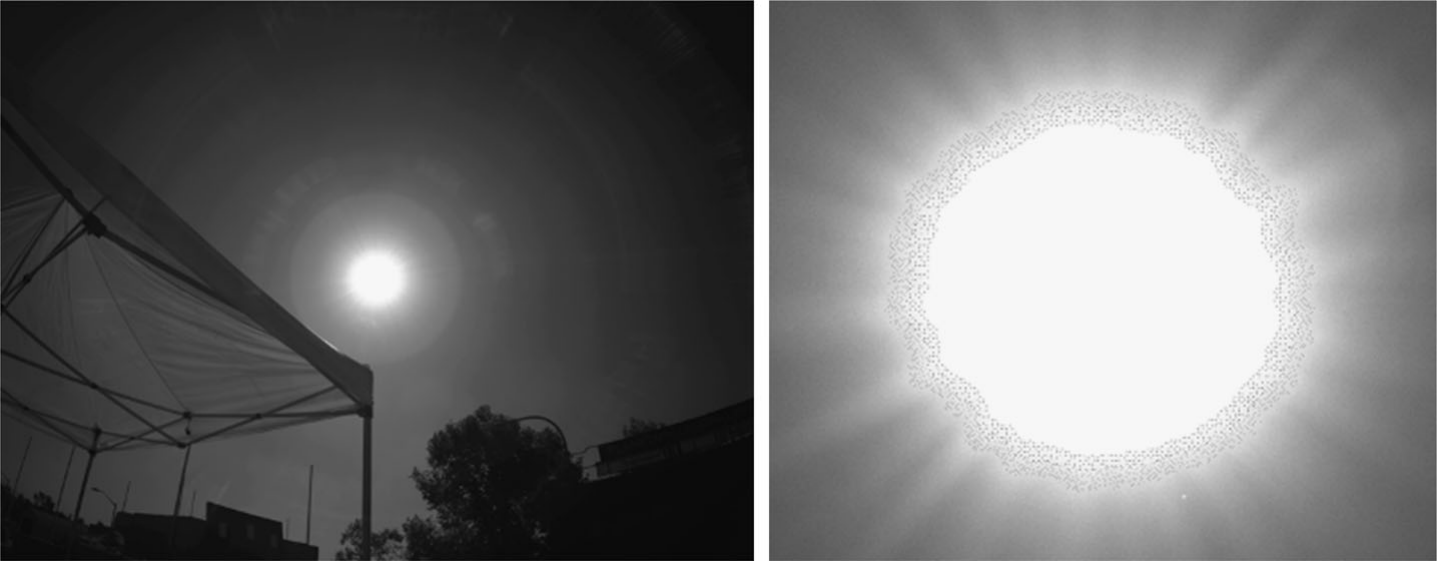
Examples of Navcam outdoor solar image testing, conducted with a flight-like Navcam. The left image was acquired in quarter-scale ($4\times4$) mode, and the image on the right was acquired in full-scale ($1\times1$) mode. The image on the right is cropped to show detail

Navcam images of the sun are used to autonomously determine the sun location in the local Mars coordinate frame. The derived sun location is subsequently used by the rover FSW to determine the rover three-axis attitude. There are three primary methods to determine the vector to the sun with the Navcam: 1) sun find, 2) sun update, and 3) sun gaze. All three methods use a disk centroiding algorithm to identify the location of the Sun in a Navcam image. A preprocessing algorithm identifies the brightest area in the image (see Fig. 34) over which to search for the sun centroid.

A sun find is requested when the location of the sun is poorly known and multiple Navcam images may be required to perform a sky search and locate the sun. A sun update is typically performed when the sun location is approximately known. A sun update uses the estimated sun vector to acquire an image of the Sun, refine the sun position and improve the rover azimuth knowledge based on the results. Finally, a sun gaze is used when the sun is at a high elevation. A sun gaze acquires multiple images of the sun over a specified time period to derive the vector of sun motion. The motion vector technique is used to avoid poor sun location solutions that may arise when the sun is near zenith.

On Mars 2020 the sun update and sungaze methods have the ability to process saturated Navcam images. When a Navcam acquires an image the sensor exposes the pixel and subtracts a background level (correlated double sampling). However, the sun is so bright that it often saturates both the exposed pixel and the background level. Subtracting these images often creates a “black sun” image that is not adequate for centroiding. To solve this problem the camera has a mode that bleeds off charge from the background signal so that it is only partially saturated. When using this mode, subtraction of the background signal from the exposed image results in an image this is properly saturated (non-zero). This causes the Sun to appear in the image as a bright spot surrounded by a ring, as shown in Fig. 34. Despite the ring artifacts the centroiding algorithm achieves high enough accuracy to meet the requirements and reduce the rover pointing error even at high sun elevations.

#### ATLO

**Fig. 35:**
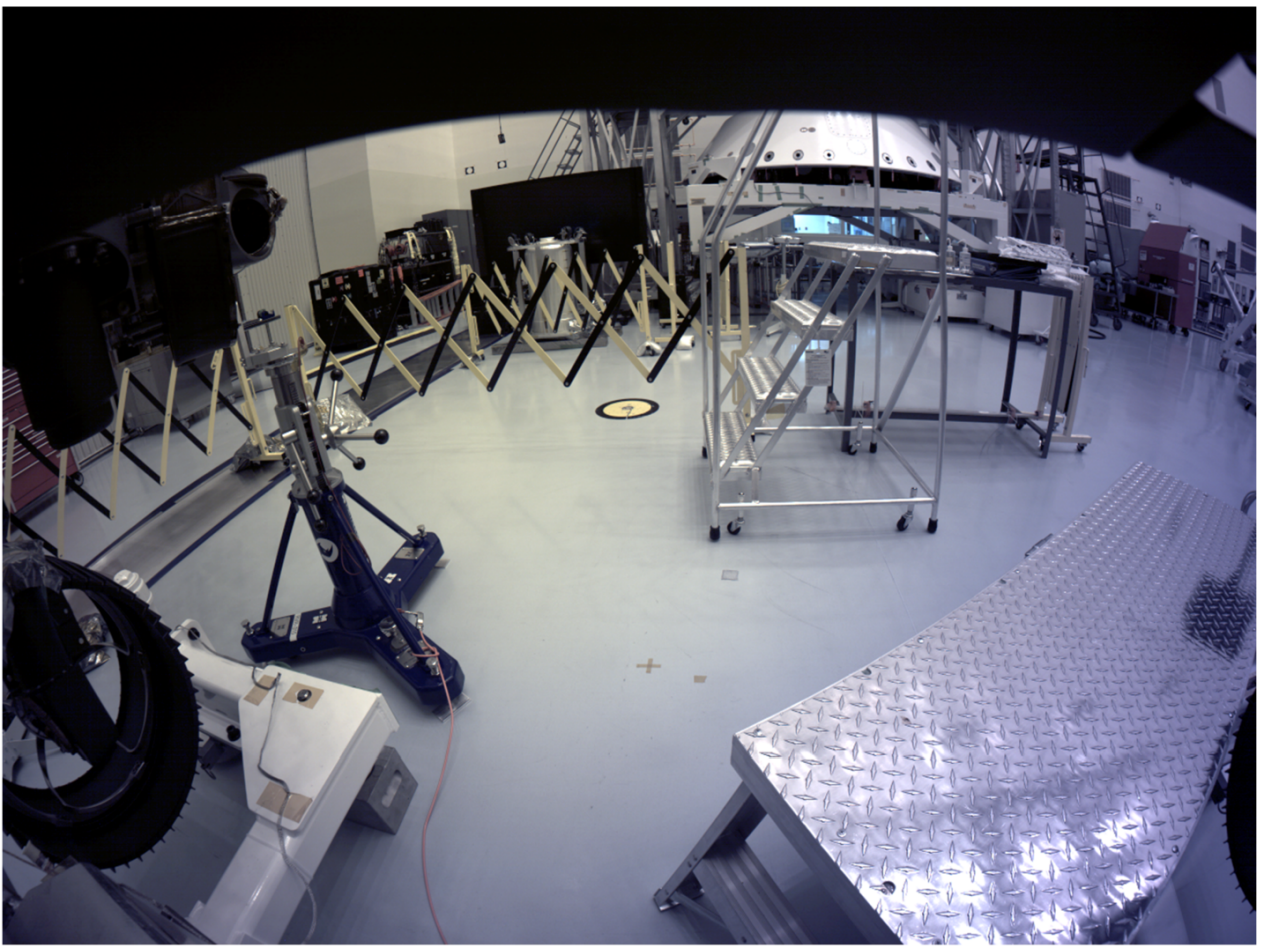
Front Left Hazcam Image. Note the top of the image is obscured by a sun visor. During operations the sun visor region of the image will not typically be returned

**Fig. 36:**
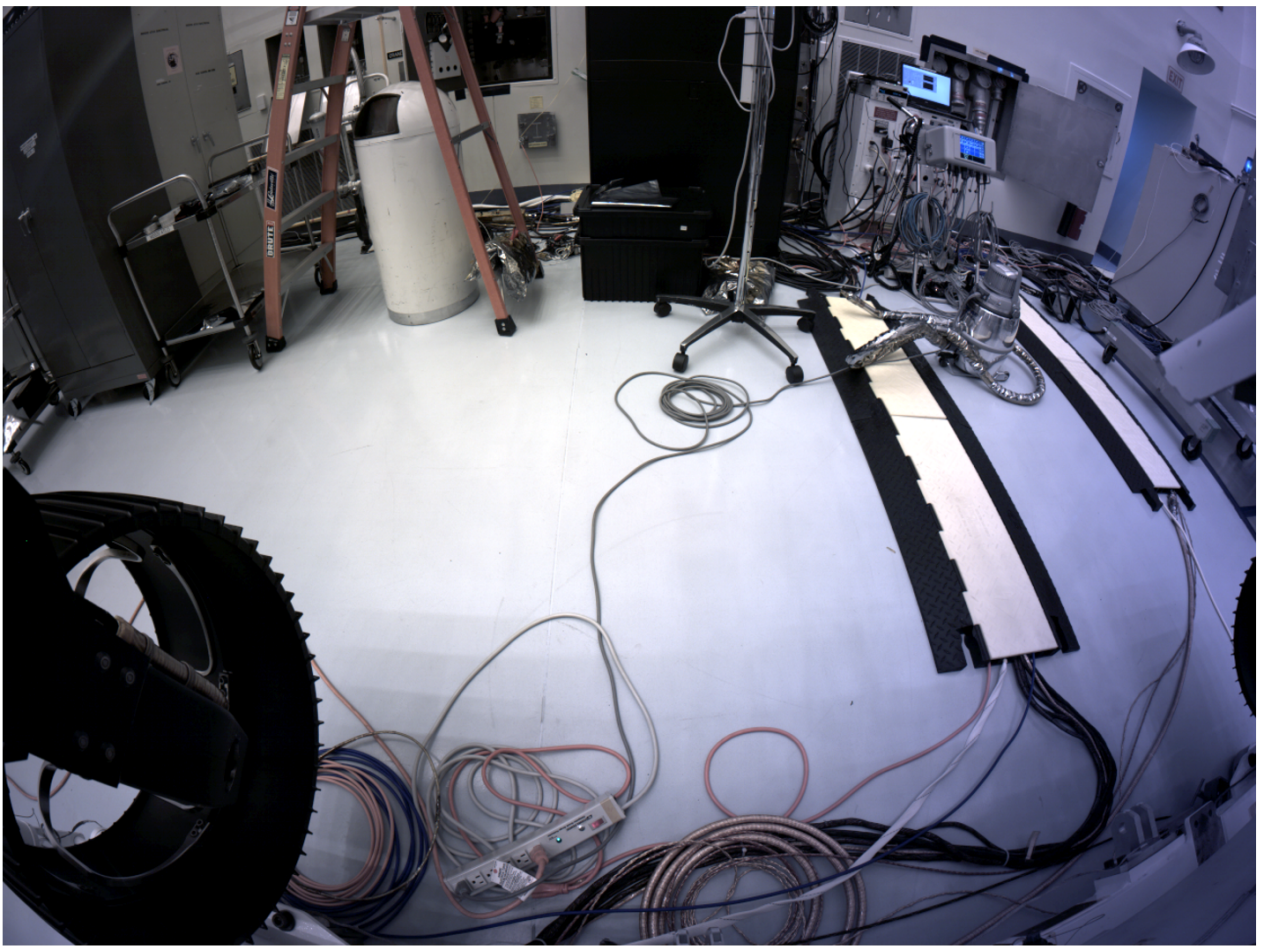
Rear Hazcam image

**Fig. 37:**
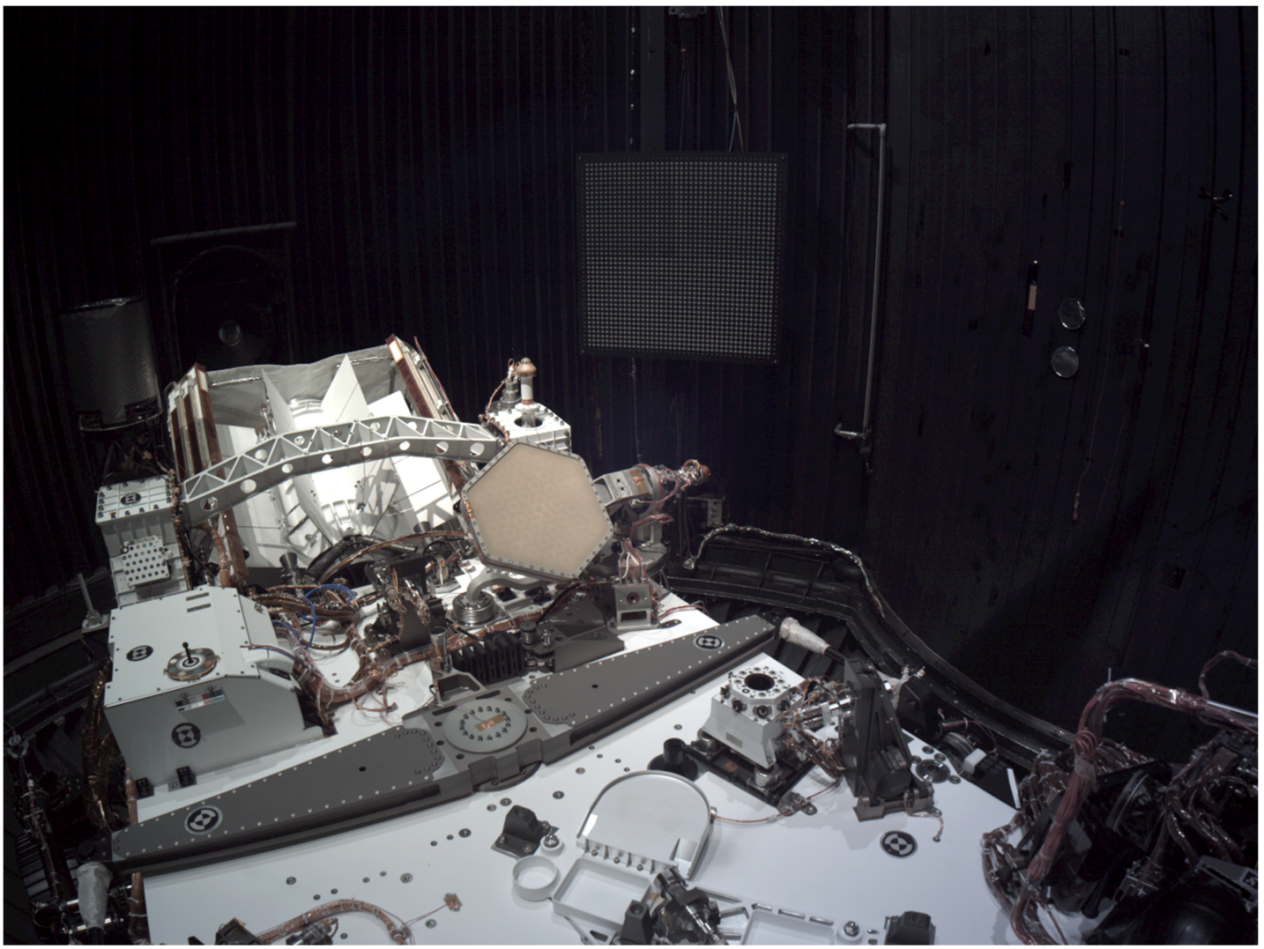
Navcam image acquired during System Thermal Vacuum testing

**Fig. 38:**
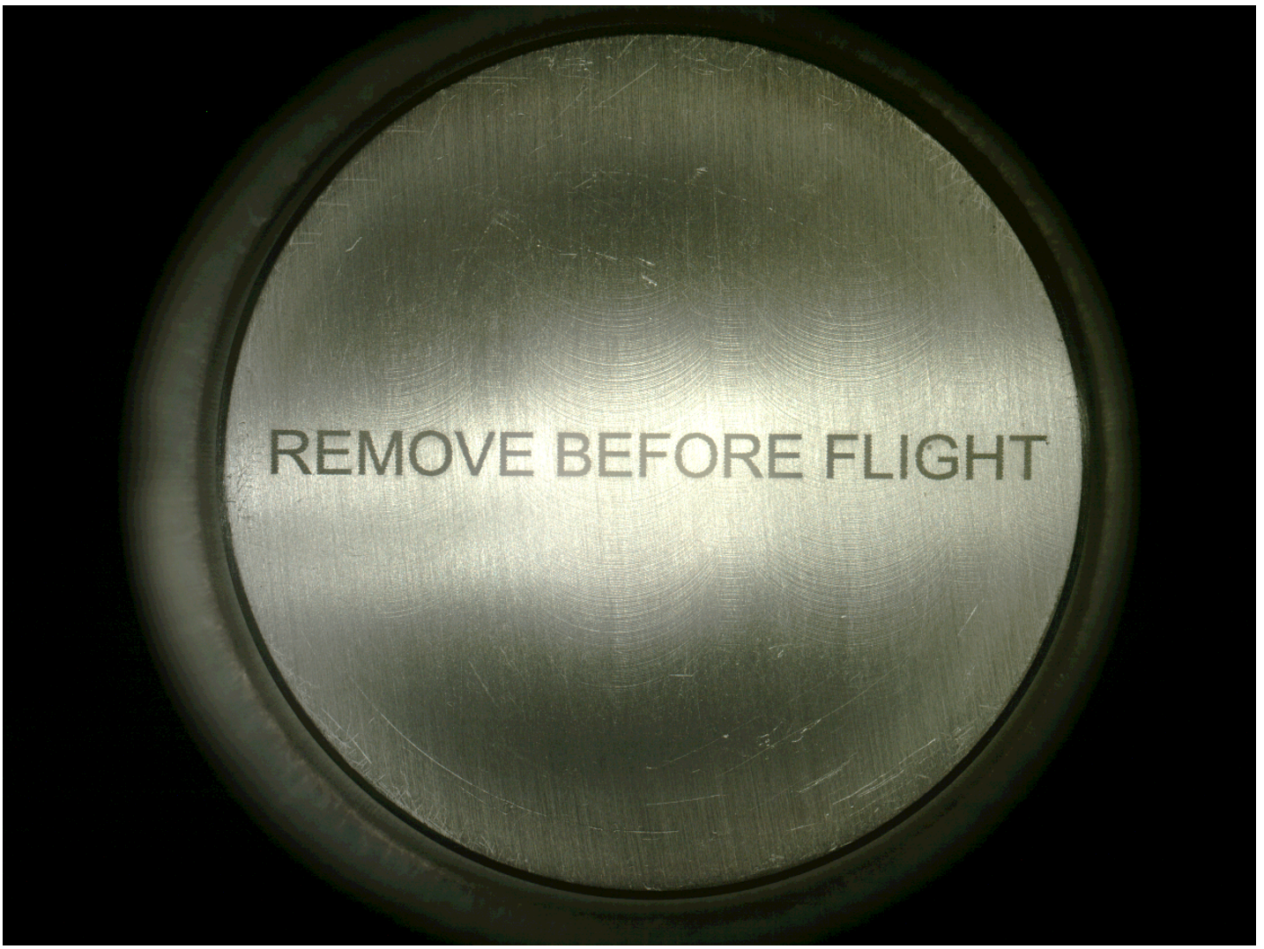
Flight Cachecam image acquired during ATLO testing. The remove-before-flight cover shown in this image has a diameter of approximately 46.4 mm. The spatial scale of this image is approximately 12.5 microns/pixel (the cover is not exactly at the best focus distance)

After the flight cameras were mounted on the rover in ATLO the locations and relative alignments of the ECAM cameras were measured in the rover navigation coordinate frame by performing a machine vision calibration similar to the calibration performed on the MER and MSL cameras (Maki et al. 2003, and Maki et al. 2012). The results of the geometric calibration are recorded in the CAHVORE camera model system of Yakimovsky and Cunningham (1978) and Gennery (2001, 2006). The cameras also participated in functional tests, including System-level Thermal Testing (STT) and other system testing, where a total of over 6,000 ECAM images (tiles) were acquired. Figures 35, 36, 37 and 38 show example images from these functional tests.

### EDLCAMs

**Fig. 39:**
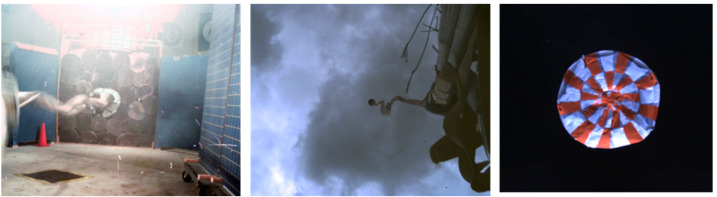
EDLCAM testing. Indoor mortar firing test (left), outdoor mortar firing test (center), and simulated parachute image testing (right)

The EDLCAMs underwent extensive functional testing prior to delivery to ATLO. Video tests were performed with live mortar firings (Fig. 39), demonstrating the ability of the DSU and cameras to acquire video of high-speed external events. After the EDLCAMs were integrated onto the rover in ATLO, they participated in functional and system testing, including EDL test runs.

### LCAM

**Fig. 40:**
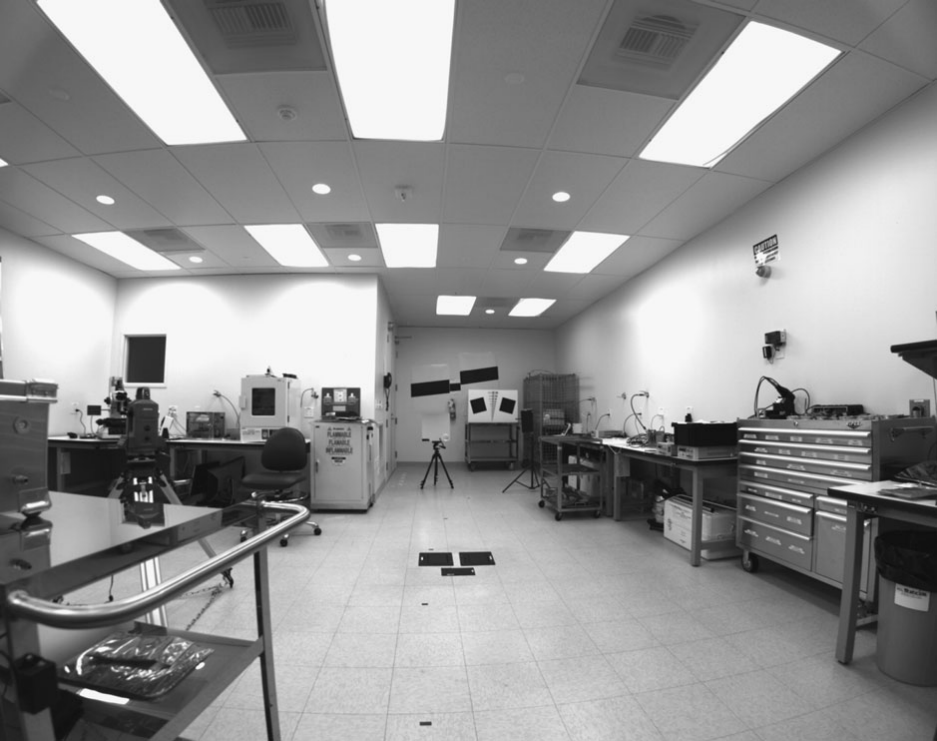
An MTF test image taken with the LCAM flight unit in the MSSS Cleanroom. This image was acquired unsummed and over the full format of the detector

**Fig. 41:**
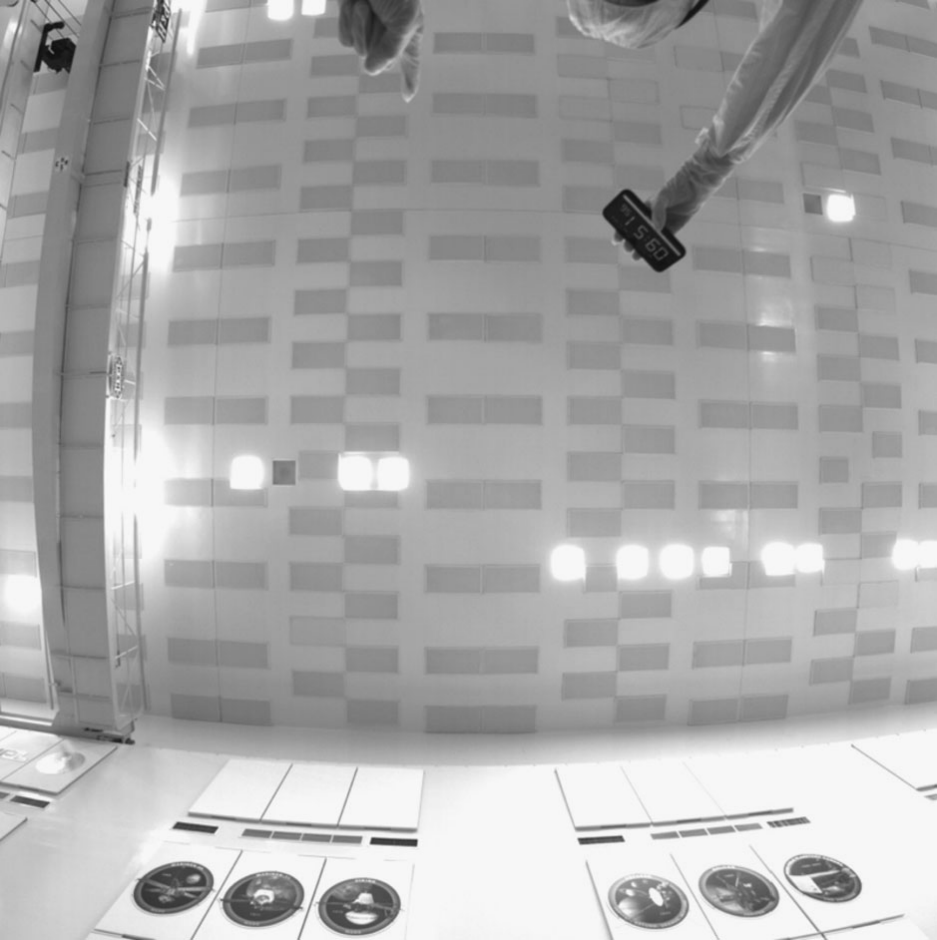
LCAM image of the ceiling in the JPL Spacecraft Assembly Facility (SAF) taken after integration with the flight rover. This image was acquired in the flight mode ($2\times2$ summed with windowing to give a $1024\times 1024$ pixel format)

Prior to delivery, LCAM was calibrated using a typical flow to measure its radiometric response, geometric properties, and behavior over temperature. An MTF test image from this testing is shown in Fig. 40. An extensive program of realistic imaging testing was performed using flight-like EM hardware imaging from a helicopter (Johnson et al. 2020). After integration with the spacecraft in ATLO, imaging testing was performed with the flight LCAM to verify functionality in the integrated system (Fig. 41) and to determine LCAM pointing in the rover coordinate system.

## Operations and Data Archiving

Operations of the ECAMs and EDLCAMs will be performed by the ECAM operations team at JPL in Pasadena, CA. The operations team members, software tools, and processes draw on heritage from the MER and MSL operations teams.

### ECAMs

**Fig. 42:**
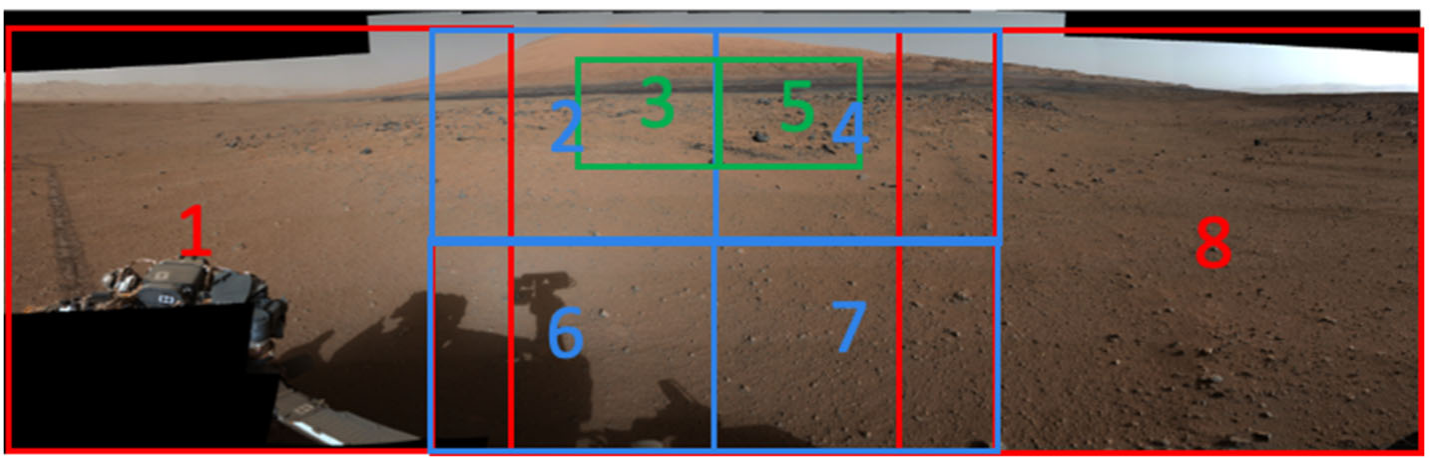
Notional Navcam operations sequence for a rover “drive direction” planning panorama, acquiring nine Navcam images of varying pixel scale. The background image for this figure (and Fig. 43) is from an MSL Mastcam panorama (Malin et al. 2017)

**Fig. 43:**
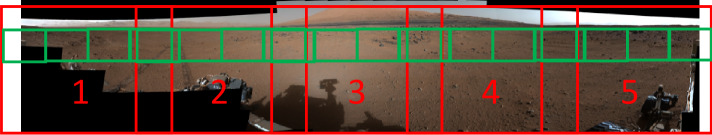
Notional Navcam $360^{\circ}$ survey panorama, comprised of a $5\times1$ quarter-scale portion (images 1 through 5, shown in red), and a full-scale $360^{\circ}$ inset panorama ($16\times1$ full-scale tiles, shown in green)

The ECAM hardware modes described in Sect. 3.1.6 allow operators to request images of differing pixel scales from the same 20 Megapixel source image. This new capability allows higher-scale image tiles to be inset into lower-scale tiles, offering a combination of wider coverage at lower scale for context and higher scale tiles targeted on particular areas of interest. This context/targeted strategy can be used to significantly reduce overall data volume. Figure 42 shows how this capability might be used for a drive direction planning panorama. The Fig. 42 example includes two quarter-scale frames (1 and 8), 4 half-scale frames (2, 4, 6, and 7), and 2 full-scale frames (3 and 5). Because images 2 through 7 all come from the same original source image, no repointing is required, although re-exposure could occur on frames 2 through 7 if optimal exposure was desired on all frames. The example in Fig. 43 shows how the multi-scale capability might be used for a $360^{\circ}$ survey panorama. In that example the farther field terrain covered by the full-scale tiles will have a spatial scale more comparable to the near-field spatial scale covered by the lower-scale tiles.

### EDLCAMs

EDLCAM operation during the surface phase of the mission will be focused primarily on the cataloging and prioritization of the over 15,000 images expected to be acquired during EDL. During the early phase of the mission, low-resolution videos, audio files, and still frame images will be downlinked. After review and analysis by the EDL teams, higher-resolution data may be requested and subsequently downlinked over the course of the surface mission. EDLCAM data will also be available to the science teams.

### LCAM

The LCAM takes images at variable rates during descent to reduce data storage requirements. Images are taken at 0.3 Hz starting just prior to heat shield separation down to the start of localization. During localization, which occurs between 4200 meters and 500 meters, images are taken at around 1 Hz. From 500 meters to the surface, images are taken at 0.3 Hz. All images will be downlinked to Earth later on during the surface mission for post-EDL analysis and assessment. These images (along with the RDC images) will be made available to the science team for potential landing site topography studies using structure-from-motion techniques (Garvin et al. 2017, 2019) or other investigations. After landing, LCAM image acquisition will not be possible because the camera interface FPGA will be reconfigured to support stereo vision and visual odometry processing tasks.

### Archiving

All of the raw images from the *Perseverance* engineering cameras will be archived in the NASA Planetary Data System (PDS) within 6 months of receipt of the data on Earth. Additionally, all of the derived geometric, radiometric, and stereo data products from the Navcams and Hazcam cameras will be also be archived, along with additional derived products from the other engineering cameras. Microphone data will also be archived.

## Summary

The *Perseverance* rover carries a next-generation imaging system that will improve the operational capabilities of the Mars 2020 mission. EDLCAM video of key EDL events will document the performance of the Mars 2020 system and inform the design for future EDL systems. The LVS/LCAM system will enable more targeted landing capabilities. Recorded audio from the rover microphone may reveal new acoustic signatures that were unknown prior to Mars 2020. If the microphone continues to operate during the surface mission, recorded sounds of the rover mechanisms may have diagnostic value for assessing the state of rover hardware. The next-generation Navcams and Hazcams will acquire images of Mars with wider fields of view and higher pixel scale than versions on previous missions. The Cachecam will acquire 12.5 micron/pixel images of the cached samples in the sample tubes. Images from the cameras will play an important role during the operational phase of the mission and will become part of the permanent record of the *Perseverance* mission. These same images will also become a key component of any future sample return mission.
